# The HIV Genomic Incidence Assay Meets False Recency Rate and Mean Duration of Recency Infection Performance Standards

**DOI:** 10.1038/s41598-017-07490-4

**Published:** 2017-08-07

**Authors:** Sung Yong Park, Tanzy M. T. Love, Lucy Reynell, Carl Yu, Tina Manzhu Kang, Kathryn Anastos, Jack DeHovitz, Chenglong Liu, Kord M. Kober, Mardge Cohen, Wendy J. Mack, Ha Youn Lee

**Affiliations:** 1 Department of Molecular Microbiology and Immunology, Keck School of Medicine, University of Southern California, Los Angeles, CA United States; 2 Department of Biostatistics and Computational Biology, University of Rochester School of Medicine and Dentistry, Rochester, NY United States; 3 Department of Medicine, and Epidemiology & Population Health, Albert Einstein College of Medicine and Montefiore Medical Center, Bronx, NY United States; 4 Department of Medicine, SUNY Downstate Medical Center, Brooklyn, NY United States; 5 Department of Medicine, Georgetown University, Washington, DC United States; 6 Department of Physiological Nursing, University of California San Francisco, San Francisco, CA United States; 7 Department of Medicine, Stroger Hospital, Chicago, IL United States; 8 Department of Preventive Medicine, Keck School of Medicine, University of Southern California, Los Angeles, CA United States

## Abstract

HIV incidence is a primary metric for epidemic surveillance and prevention efficacy assessment. HIV incidence assay performance is evaluated via false recency rate (FRR) and mean duration of recent infection (MDRI). We conducted a meta-analysis of 438 incident and 305 chronic specimens’ HIV envelope genes from a diverse global cohort. The genome similarity index (*GSI*) accurately characterized infection stage across diverse host and viral factors. All except one chronic specimen had *GSI*s below 0.67, yielding a FRR of 0.33 [0-0.98] %. We modeled the incidence assay biomarker dynamics with a logistic link function assuming individual variabilities in a Beta distribution. The GSI probability density function peaked close to 1 in early infection and 0 around two years post infection, yielding MDRI of 420 [361, 467] days. We tested the assay by newly sequencing 744 envelope genes from 59 specimens of 21 subjects who followed from HIV negative status. Both standardized residuals and Anderson-Darling tests showed that the test dataset was statistically consistent with the model biomarker dynamics. This is the first reported incidence assay meeting the optimal FRR and MDRI performance standards. Signatures of HIV gene diversification can allow precise cross-sectional surveillance with a desirable temporal range of incidence detection.

## Introduction

HIV incidence, the number of individuals newly-infected within a given time (1~2 years), is a key measure of the epidemic’s rise and decline^1^. Importantly, it serves as a direct metric of HIV intervention and prevention trial efficacy, providing timely feedback for intervention programs and guiding resource allocation and government responses^2–4^. Developing reliable assays to distinguish recent from chronic infections has been a long-standing goal of the HIV community^5–9^. In particular, cross-sectional population sampling via a single blood draw has been considered to be the ideal platform to determine HIV incidence.

HIV incidence assay performance is evaluated by each assay’s mean duration of recent infection (MDRI) and false recency rate (FRR)^10^. The MDRI is the average length of time in which subjects are classified as recently infected by an assay, and the FRR is the probability that a chronically infected subject is misclassified. A low FRR is required for precise incidence determination and a higher MDRI allows incidence to be estimated from a smaller cross-sectional survey sample size^2, 11^. The Consortium for the Evaluation and Performance of HIV Incidence Assays (CEPHIA) reported MDRIs of serological HIV incidence assays to range from 188 to 333 days with FRRs between 1.3-9.7%^12^, and recently introduced viral load criteria to improve estimates^13^. Other studies^14–16^ reported FRRs ranging from 0% to 10.2% and MDRIs from 50 to 276 days, and a high misclassification rate in subjects with low CD4 T cell counts^16^, underachieving the optimal performance standards (MDRI ~ 1 year and FRR <1%)^17^. Multi-assay algorithms^13^ have been proposed to meet the minimal performance standards (MDRI ~ 4 months and FRR <2%)^17^.

As an alternative to serological approaches, which assess host signals, virus signals have shown great promise for the precise assessment of HIV incidence^18–20^; incident infections are recognized by identification of closely related gene sequences within the HIV population of an infected individual. One or more clusters of similar strains indicate that a viral population has recently evolved from either a single or multiple transmitted viruses. In contrast, during chronic infection, viral strains diversify within an individual as a result of mutations accumulated via HIV reverse transcriptase errors, recombination and immune selection. The presence of closely related strains as a signature of recent infection is detected using genomic biomarkers including the genome similarity index (*GSI*)^18, 19^.

Here we comprehensively evaluate the genomic assay with a globally representative population to assess its cross-sectional survey application. To our knowledge this is the first study to measure a genomic assay’s FRR and MDRI. Combining meta-analysis of infected individuals with well-characterized duration of infection, new sequencing of longitudinally followed subjects and statistical modeling, we investigate whether genomic screening can meet performance standards for incidence determination.

## Results

**Figure 1:**
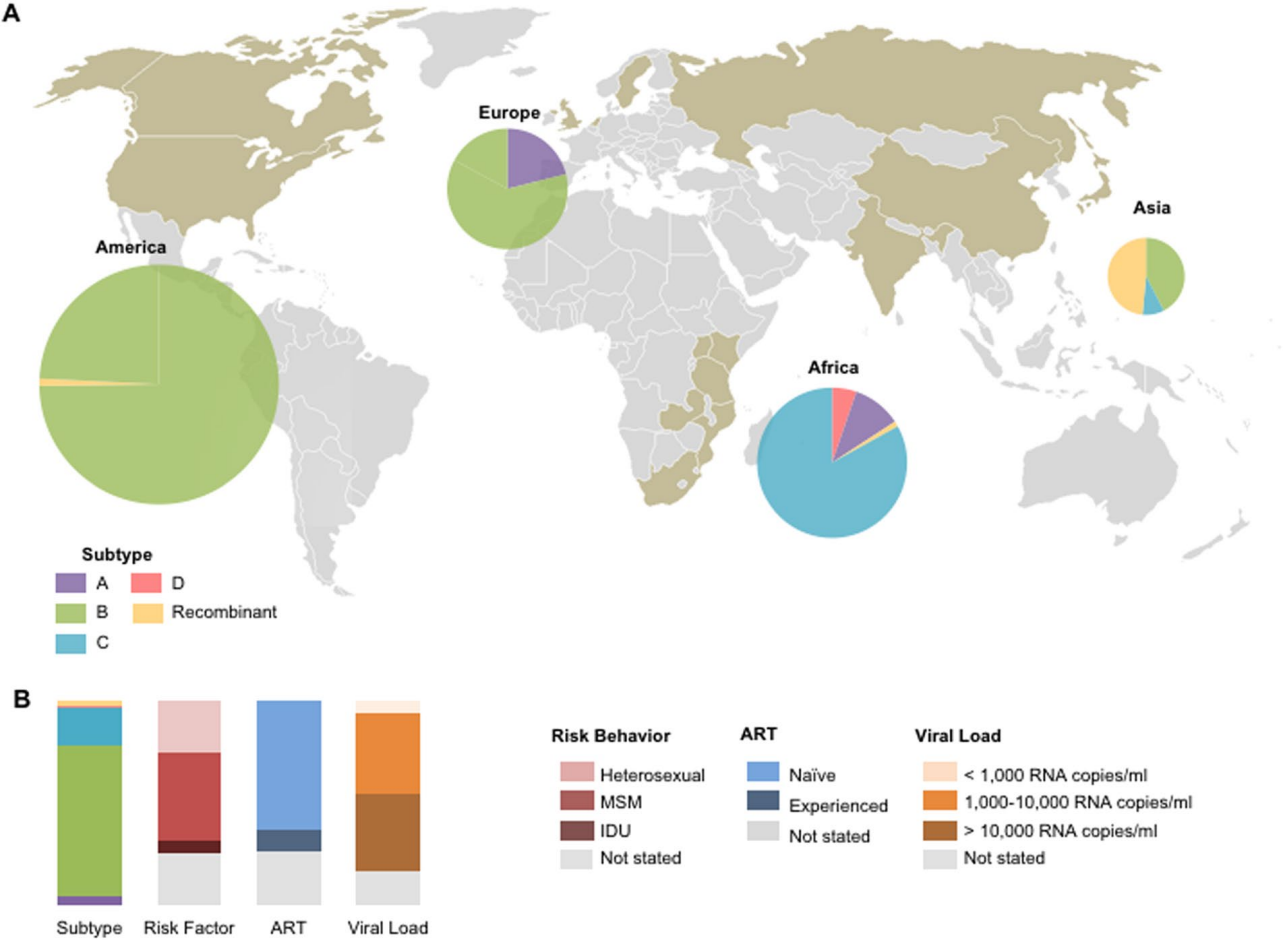
Global cohort characteristics (**A**). Geographic and subtype distribution of 805 specimens from the shaded (taupe) countries. These specimens are further described in Tables 1 and S1–S5. Pie charts indicate the subtype of included specimens, where the diameter denotes the proportional representation of each continent to the total specimen. The map was generated by Microsoft PowerPoint (Version 15.30) using a template available at https://commons.wikimedia.org/wiki/File:Color_world_map.png which is released into the public domain at Wikimedia Commons, free media repository. (**B**). The profiles of the 805 specimens’ subtype, risk behaviors, ART status, and viral load.

Figure 1 shows the geographic source of the 805 specimens analyzed in this study. The genomic incidence assay’s FRR and MDRI were measured using this cohort in which infection duration was well-characterized using documented HIV negative and positive dates, Fiebig stage, or specimen collection interval. This global population represents both sexes and a wide range of viral subtypes, risk behaviors, ART experiences, viral loads, CD4 T cell counts and durations of infection, as presented in Tables S1–S5 (see Methods).

We collected and newly sequenced HIV envelope gene segments spanning part of V3 and entire V4 (HXB2 7134-7499). HIV envelope gene sequences have provided a more informative signature for incidence detection, as compared to HIV gag gene sequences^18, 19, 21^. We calculated each specimen’s incidence biomarker, the genome similarity index (*GSI*), as previously described^19^,

1 $$GSI=\frac{2}{n(n-1)}\sum _{d=0}^{3}\{\sum _{i=1}^{n}\sum _{j=i+1}^{n}I(H{D}_{ij}=d)\},$$

which quantifies the proportion of sequence pairs that differ by up to 3 nucleotide bases (Hamming Distance (HD) = 3). Here *n* is the number of sequences and $I(H{D}_{ij}=d)$ is an indicator function. Recent infections would have a greater *GSI* than chronic infections due to the similarity of sequences within each transmitted virus lineage^18, 19^.

**Figure 2:**
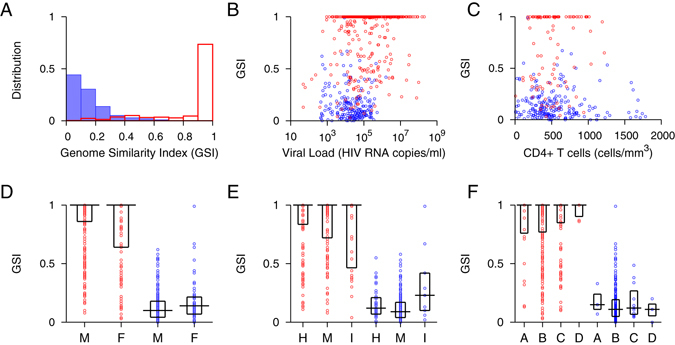
Genome Similarity Index (*GSI*) of incident and chronic infections. (**A**) The *GSI* distribution of 438 incident specimens is presented in red boxes and that of 305 chronic specimens in blue. The 305 chronic specimens include 274 chronic specimens listed in Table S1, 8 chronic specimens from the longitudinal cohort in Table S3 and 23 chronic specimens from the WIHS cohort in Table 1. The 438 incident specimens consist of 252 single time point incident specimens in Table S2 and 186 incident specimens from the longitudinal cohort in Table S3. All chronic specimens were collected from subjects documented to have been HIV-1 infected for over two years, and all incident specimens were collected within 2 years of HIV-1 infections, according to Fiebig staging and sampling intervals. The two distributions were clearly polarized; the majority of incident subjects had GSIs above 0.9, while all chronic subjects except one had GSIs below 0.67. (**B**) *GSI* and viral load for incident (red) and chronic (blue) specimens where viral load was available. Viral load did not significantly correlate with 207 chronic specimens’ *GSI* (Spearman’s correlation *ρ* = −0.069 and p = 0.32) but associated with 433 incident *GSI* (Spearman’s correlation *ρ* = 0.17 and p < 0.01) although, as indicated by a small correlation coefficient, this association was weak. (**C**) *GSI* and CD4 + T cell count where available were not statistically correlated in either 104 incident (red) or 209 chronic (blue) specimens (Spearman’s correlation *ρ* = 0.12 and p = 0.24 and *ρ* = −0.11 and p = 0.11, respectively). (**D**) *GSI* of male (M) and female (F) incident (red) and chronic (blue) specimens. Box plots represent median and first and third quartiles. Incident specimen’s *GSI* was not sensitive to sex (299 male vs. 142 female, Wilcoxon rank sum test, p = 0.22), but chronic *GSI* was sensitive (Wilcoxon rank sum test, p = 0.024), presumably due to unbalanced sample size (226 male vs. 55 female) as suggested by overlapping quartiles. In a permutation test, this association was nonsignificant (p = 0.076). (**E**) *GSI* of incident (red) and chronic (blue) specimens from different risk groups (H: heterosexual, M: men who have sex with men, I: intravenous drug user). Incident *GSI* was not sensitive to risk behavior (156 heterosexual vs. 143 MSM vs. 34 IDU, Kruskal-Wallis tests, p = 0.094), but chronic *GSI* was sensitive (Kruskal-Wallis test, p = 0.015), likely due to unbalanced sample size (46 heterosexual vs. 201 MSM vs. 9 IDU). The p-value was 0.009 in a permutation test. (**F**) *GSI* for incident (red) and chronic (blue) specimens of subtype A, B, C, and D. Neither incident (31 subtype A, 279 subtype B, 134 subtype C, and 6 subtype D) nor chronic (3 subtype A, 280 subtype B, 11 subtype C, and 3 subtype D) GSIs differed significantly among subtypes (Kruskal-Wallis test, p = 0.61 and p = 0.70, respectively).

Figure 2A compares the genomic biomarker profile of 305 chronic infections with documented HIV infection exceeding 2 years to that of 438 recent infections acquired within 2 years. The vast majority of incident subjects had *GSI*s above 0.9. However, the *GSI* of the remaining incident subjects was widely distributed, as shown in Fig. 2A. All except one chronic subject had *GSI* values below 0.67 and thus we designated the biomarker cutoff as 0.67, yielding a false recency rate (FRR) of 0.33% [0–0.98%]. Here the FRR’s 95% CI was obtained by a resampling method. When we sampled subjects with replacement by considering that 305 chronic specimens were collected from 147 subjects (Table 1 and Table S1), we obtained a FRR of 0.68% [0–2.0%]. The FRR was considerably smaller than that of any currently available incidence assay including the limiting antigen assay (LAg), 1.3% [0.3–3.2%]^12^. The genomic assay’s low FRR was obtained from a diverse cohort including subtype D viruses and ART-experienced subjects (Fig. 1). Importantly, this FRR met the current performance guideline of less than 1%^17^. When we examined the sensitivity of the genomic biomarker to viral and host factors, overall, our biomarker remains relatively robust across viral and host specific factors (Fig. 2B–F).

**Table 1:** WIHS specimens’ infection duration and viral and host factors.

Subject	Visit	Documented Infection Duration (Days)^a^	Subtype	Risk Behaviour	ART status	Viral Load (RNA copies/ml)	CD4 Count (cells/mm^3^)	Number of Sequences
TY2947	18	[81–251]	B	NS	Naive	920	967	14
	22	[732–902]	B	NS	Naive	6400	767	15
	24	[1105–1275]	B	NS	Naive	8500	631	12
RH7057	3	[0–146]	B	NS	Naive	14000	700	17
	4	[188–334]	B	NS	Naive	15000	252	14
	5	[384–530]	B	NS	Experienced	1592	328	13
	7	[733–879]	B	NS	Experienced	4800	374	10
	10	[1126–1272]	B	NS	Experienced	3200	359	11
IS5366	18	[1085–1945]	B	NS	Naive	1100	125	13
GE6106	9	[0–183]	B	IDU	Naive	NS	804	18
	10	[182–365]	B	IDU	Naive	230	510	10
	11	[350–533]	B	IDU	Naive	190000	576	13
	13	[728–911]	B	IDU	Naive	45000	552	17
	15	[1133–1316]	B	IDU	Naive	160000	431	13
UN2011	22	[1079–2123]	B	IDU	Experienced	3800	458	12
OY9080	17	[0–187]	B	Heterosexual	Naive	NS	NS	10
	18	[183–370]	B	Heterosexual	Naive	50000	585	11
	19	[357–544]	B	Heterosexual	Naive	45000	573	11
	21	[723–910]	B	Heterosexual	Naive	48000	319	11
	23	[1087–1274]	B	Heterosexual	Naive	34000	308	11
DA1342	2	[0–176]	B	NS	Naive	12000	584	11
	3	[160–336]	B	NS	Naive	20000	539	14
	4	[417–593]	B	NS	Naive	10000	416	16
	6	[789–965]	B	NS	Naive	1505	359	10
	8	[1070–1246]	B	NS	Naive	1900	367	11
SI1392	3	[0–134]	R-A/G	NS	Naive	1280	760	16
	5	[431–565]	R-A/G	NS	Naive	37000	232	8
	7	[739–873]	R-A/G	NS	Experienced	1148	357	12
	9	[1028–1162]	R-A/G	NS	Experienced	4300	227	11
	10	[1182–1316]	R-A/G	NS	Experienced	6100	245	8
CC2874	23	[1103–1263]	B	NS	Naive	11000	252	15
JG8930	28	[0–237]	B	NS	Naive	11000	908	14
JY3080	33	[0–183]	B	NS	Naive	NS	NS	10
	34	[182–365]	B	NS	Naive	101925	940	14
PR4290	30	[879–1089]	B	NS	Naive	1530	368	13
XE9655	23	[0–176]	B	NS	Naive	NS	NS	11
	28	[844–1020]	B	NS	Naive	36000	444	12
FQ2419	16	[756–2638]	B	IDU	Experienced	47000	327	19
	18	[1134–3016]	B	IDU	Experienced	32000	301	11
DV6934	2	[0–245]	B	IDU	Naive	NS	377	15
NM1689	6	[0–173]	B	IDU	Naive	39000	836	10
	9	[594–767]	B	IDU	Experienced	53000	920	10
	10	[775–948]	B	IDU	Experienced	12000	646	10
	12	[1197–1370]	B	IDU	Experienced	1600000	658	10
SS0342	9	[0–177]	B	IDU	Naive	210000	352	15
	10	[160–337]	B	IDU	Naive	15000	487	12
	11	[354–531]	B	IDU	Experienced	3500	614	14
	13	[720–897]	B	IDU	Experienced	8800	325	12
	16	[1292–1469]	B	IDU	Experienced	8700	173	15
BQ7042	17	[1035–1267]	B	IDU	Experienced	9400	630	13
VE6375	25	[0–127]	B	IDU	Naive	16000	403	13
	27	[414–541]	B	IDU	Naive	140	260	15
EJ7211	14	[0–188]	B	Heterosexual	Naive	NI	909	12
	16	[358–546]	B	Heterosexual	Naive	2600	1220	11
	18	[699–887]	B	Heterosexual	Naive	140000	1449	9
	20	[1044–1232]	B	Heterosexual	Naive	33000	683	14
TI9054	3	[0–176]	B	NS	Naive	5800	795	18
	4	[166–342]	B	NS	Naive	19000	493	12
	5	[349–525]	B	NS	Naive	17453	427	12

The minimum duration of infection of each individual was the elapsed time between the date of first HIV positive documentation and the date of specimen collection. The maximum duration of infection was the time between the last HIV negative test and blood sample collection. NS, not stated ; Naive, subject has not received ART before this time point; Experienced, patient is currently or has previously received ART; IDU, intravenous drug user.

**Figure 3:**
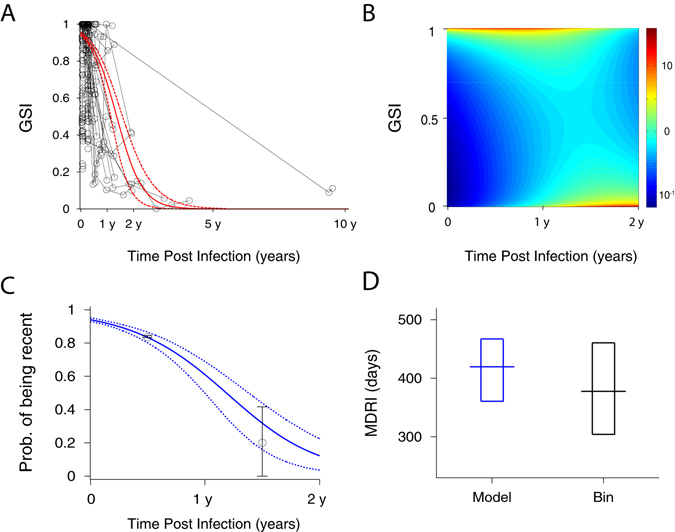
Genomic biomarker dynamics over time. (**A**) *GSI* dynamics of 194 longitudinal and 252 single time point incident specimens along with the beta distribution model fit. Forty three subjects in Table S3 were serially followed from Fiebig stage I-V (circles with black solid lines) and 252 single time point incident specimens were collected at Fiebig stage I-V (circles), as presented in Table S2. The *GSI* varies between individuals, but in the majority of cases is close to one for new infections and drops towards zero over time. The average biomarker dynamics were modeled by logistic link function and the variation between individuals was modeled by the beta distribution as in Eqs (2–3). The best fit of the model for the average *GSI* dynamics (solid red curve) and its 95% confidence intervals (CI) (dashed red curves) are presented. The maximum likelihood estimates of the model parameters are *M* = 495.8 [415.1–575.6], *S* = 176.8 [124.3–239.7], *V* = 0.96 [0.86–1.12] and *c* = 0.95 [0.94–0.96]. Each parameter’s 95% CI was obtained by resampling incident specimens’ biomarker data 10,000 times. The average biomarker’s 95% CI (dashed red curves) is the 95% CI of 10,000 fitted biomarker dynamics curves for each time point. (**B**) The density plot of the estimated *GSI* distribution over time. The *GSI* probability density function peaked (red) close to 1 during early infection and at 0 around two years post infection. However, around one year the density function peaked in both high and low *GSI* regions. These profiles collectively reflect the sequence data trends at the population level. The model estimate of the probability of being recent, defined in Eq. (4), is presented by a blue line and the proportion of subjects with *GSI* greater than the threshold in each one year bin is presented by black circles. The 95% CIs are presented by blue dashed curves and black lines, respectively. The beta distribution model was consistent with the one year bin evaluation. (**D**) The MDRI estimated by the model (blue), 420 [361–467] days, was compared with that from the bin-method (black), 378 [304–460] days.

Figure 3A plots the *GSI* of 194 longitudinal and 252 single time point incident specimens as a function of estimated days post infection; *GSI* is close to one for new infections and drops towards zero over time as the intrahost HIV population diversifies. We observed considerable variation in *GSI* values at a given time post infection, in particular around 1 year (Fig. 3A). We statistically modeled the average biomarker dynamics by assuming a Beta distribution for population-wide biomarker variability at a given time since infection. The average *GSI* as a function of time *t* since infection is

2 $$\overline{GSI}(t)=c\frac{1+\exp [-M/S]}{1+\exp [(t-M)/S]},$$

where *M*, *S*, and *c* are regression parameters. Around the mean biomarker,$\,\overline{GSI}(t)$, we assumed individual variabilities in a Beta distribution with the following *GSI* probability density function

3 $$p(GSI|t)=\frac{{\rm{\Gamma }}[\alpha (t)+\beta (t)]}{{\rm{\Gamma }}[\alpha (t)]{\rm{\Gamma }}[\beta (t)]}GS{I}^{[\alpha (t)-1]}{(1-GSI)}^{[\beta (t)-1]},$$

with $\alpha (t)=V\times \overline{GSI}(t)$ and $\beta (t)=V[1-\overline{GSI}(t)].$ Here *V* is the precision parameter. Then the probability of being recent, i.e., the probability of a sample’s *GSI* exceeding the cut-off *θ*, is given by

4 $${P}_{R}(t)=p(GSI > \theta |t)={\int }_{\theta }^{1}p(GSI|t)d(GSI)=1-{I}_{\theta }[\alpha (t),\beta (t)],$$

where ${I}_{\theta }[\alpha (t),\beta (t)]$ is the regularized incomplete beta function.

The mean duration of recent infection is defined as^12^,

5 $$MDRI={\int }_{0}^{T}{P}_{R}(t)\,dt={\int }_{0}^{T}1-{I}_{\theta }[\alpha (t),\beta (t)]dt,$$

where *T* is the cutoff between incident and chronic infections, chosen as two years^10, 11^.

We estimated the model parameters, *M*, *S*, *c* and *V* using the likelihood function

6 $$\begin{array}{rcl}L(M,S,c,V) & = & \prod _{i=1}^{{n}_{d}}{\rm{p}}(GS{I}_{i}|{t}_{i})\\  & = & \prod _{i=1}^{{n}_{d}}\frac{{\rm{\Gamma }}[\alpha ({t}_{i})+\beta ({t}_{i})]}{{\rm{\Gamma }}[\alpha ({t}_{i})]{\rm{\Gamma }}[\beta ({t}_{i})]}GS{I}^{[\alpha ({t}_{i})-1]}{(1-GSI)}^{[\beta ({t}_{i})-1]},\end{array}$$

where *n*_*d*_ is the total number of incident samples.

The fitted biomarker dynamics clearly traced the average *GSI* dynamics measured from 438 incident specimens (Fig. 3A). Importantly, the model Beta distribution also reflects the profile of *GSI* variability across individuals over time. The *GSI* probability density function peaked close to a *GSI* of 1 in early infection and at a *GSI* of 0 around 2 years post infection (Fig. 3B). Around 1 year post infection the incident subjects’ *GSIs* were bimodal, peaking at both ends of the *GSI* spectrum (Fig. 3B). We can conclude that the flexible Beta distribution adequately illustrated not only the average biomarker dynamics but also the breadth among individuals over time.

**Figure 4:**
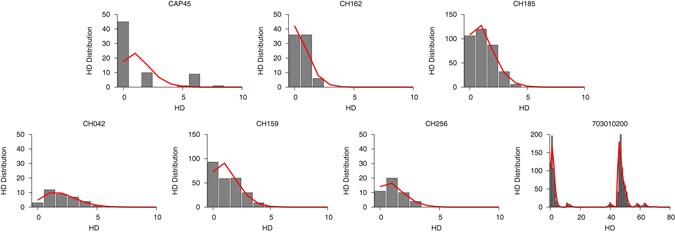
The intersequence Hamming distance (HD) distribution (grey bars) of HIV-1 full envelope gene sequences obtained from each subject in the slow (CAP45, CH162, and CH185) and fast *GSI* dynamics groups (CH042, CH159, CH256, and 703010200) along with the best fit (red curve) of the Shifted Poisson Mixture Model (SPMM)^22^. The SPMM estimated a single founder variant for all subjects except subject 703010200, whose infection was estimated to originate from six founders (when two putative recombinant strains from subject 703010200 were excluded, the number of founders was estimated to be 4. Here the minimum distance between founder variants was set as 10). The infection duration estimated by SPMM for subject CAP45, CH162, CH185 in the slow group was 24.1 [12.2–35.9] (goodness of fit p < 0.0001), 11.0 [3.4–18.6] (p = 0.07) and 21.4 [13.9–28.8] (p = 0.49) days and for subject CH042, CH159, CH256, and 703010200 in the fast group was 35.7 [19.2–52.2] (p = 0.90), 22.8 [14.4–31.1] (p = 0.002), 21.0 [8.9–33.1] (p = 0.66) and 28.4 [22.2–34.5] (p < 0.0001) days, respectively. The SPMM fits’ sum of squared errors (SSE) and Akaike information criteria (AIC) were 0.33 (291.0), 0.024 (152.9), 0.0017 (966.7), 0.0078 (118.7), 0.022 (761.0), 0.011 (119.2), and 0.0055 (4098.1) for subject CAP45, CH162, CH185, CH042, CH159, CH256 and 703010200, respectively.

**Figure 5:**
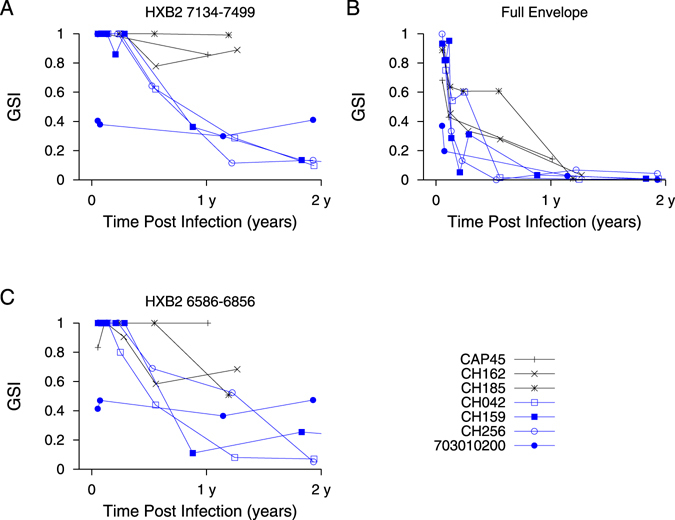
*GSI* dynamics in slow and fast decay groups. *GSI* dynamics of envelope gene segment HXB2 7134-7499 (**A**), full envelope gene (**B**), and envelope gene segment HXB2 6586-6856 (**C**) in slow (CAP45, CH162, and CH185) and fast (CH042, CH159, CH256, and 703010200) groups.

We sought to investigate why, at around one year post-infection, the *GSI* distribution peaks at 1 and 0, rather than clustering around the mean *GSI*. We compared subjects whose *GSI* remained above the incidence cutoff (*θ*) after one year (subjects CAP45, CH162, and CH185) with subjects whose *GSI* did not (subjects 703010200, CH256, CH042, CH159). We first speculated that rapid GSI decay could indicate transmission of multiple founder viruses. When we estimated the number of transmitted/founder variants using the Shifted Poisson Mixture Model^22^, only one infection (703010200) was found to originate from multiple founders, suggesting that rapid *GSI* decay could not be attributed to transmission of multiple founder variants. (Fig. 4). All individuals in the slow and fast groups were infected with subtype C virus and all except 703010200 were ART-naïve throughout the follow-up period. The risk behavior was either heterosexual or not stated, and the viral load and CD4 count did not differ statistically significantly between these groups (Wilcoson rank sum test, p = 0.52 and p = 0.44, respectively). The differences in diversification rate were also reflected in evolution of the whole envelope gene and another envelope gene segment (HXB2 6586–6856) (Fig. 5). Therefore, GSI decay speed is likely influenced by inter-subject variation in other intrahost factors, likely immune selection.

We obtained the probability of being recent *P*_*R*_(*t*) as a function of days since infection (Fig. 3C) and the resulting MDRI (Fig. 3D) using Eqs (4) and (5). The genomic assay’s MDRI was estimated to be 420 [361–467] days, which is considerably greater than any previously reported MDRI^12, 14–16^. We compared our model MDRI estimates with that from the proportion of subjects whose *GSI* values were greater than the cutoff, *θ* in a one-year bin (Fig. 3C and D). This alternative MDRI, 378 [304–460] days, was shorter but significantly overlaps with the model estimate (Fig. 3D). When we sampled subjects with replacement, the model MDRI estimate was 415 [275–534] days, and the MDRI estimated by the one-year bin method was 399 [298–678] days. One limitation of our MDRI estimate is that most incident specimens, around 90%, were obtained within 6 months of infection. Therefore, additional specimens collected between 6 months and 2 years after infection ought to be tested to improve MDRI accuracy.

**Figure 6:**
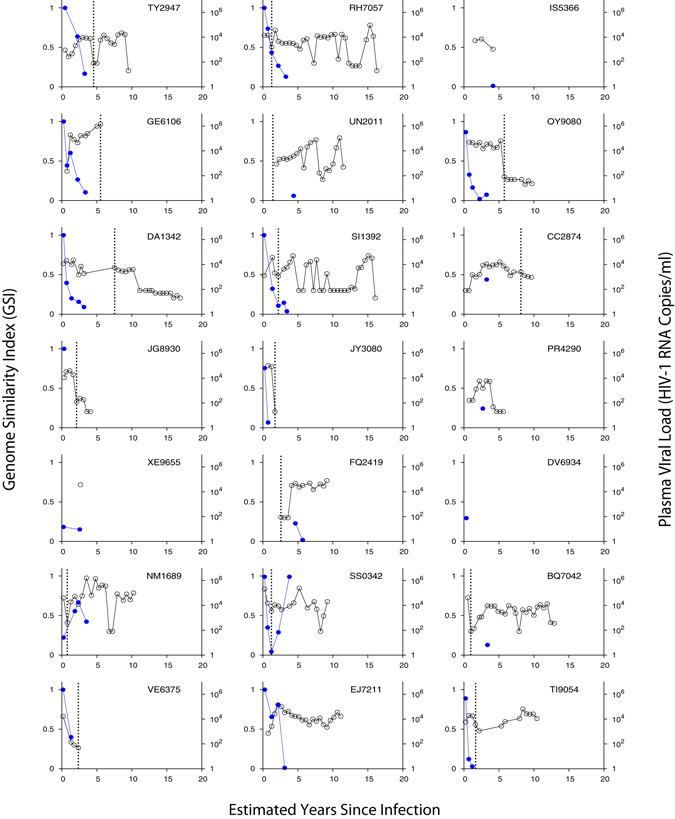
Genomic incidence assay biomarker and viral load dynamics of 21 WIHS longitudinally followed subjects. Each subject’s *GSI* is represented by blue filled dots and viral load by black empty dots. The *GSI* was measured from eight or more HIV-1 envelope gene segments (HXB2 7134-7499) from each specimen using Eq. (1). Years since infection was estimated by taking the mid-point between each subject’s last HIV negative date and first HIV positve date, and each subsequent sample collection date. The dotted black vertical line indicates ART initiation. Of 14 longitudinally sequenced subjects all except two subjects (NM1689 and SS0342) showed *GSI* decline over time as expected.

We next tested our assay by newly sequencing 744 HIV-1 envelope gene segments (HXB2 7134-7499) from 21 seroconverters identified in the WIHS cohort (see Methods for sequencing procedures). These 21 women were serially followed from HIV negative status. Each individual’s HIV negative and first positive dates allowed us to designate a precise interval for time since infection, as shown in Table 1. Figure 6 plotted viral load and *GSI* dynamics for the 21 individuals. All serially sequenced individuals except, NM1689 and SS0342, showed *GSI* decline over time. These two subjects exhibited increases and decreases in *GSI* within 5 years of infection. Of the 13 first HIV positive specimens all except one had incident *GSI* signatures, and all specimens confirmed to be collected after 2 years of infection had chronic *GSI* signatures, except the last sequenced specimen from subject SS0342 (Fig. 6).

**Figure 7:**
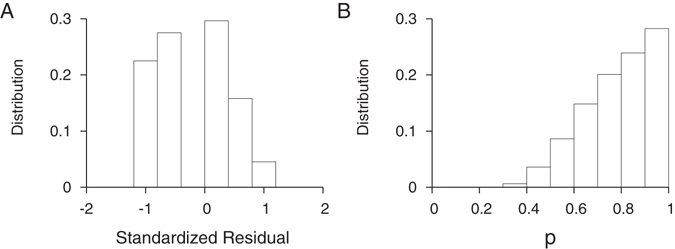
Biomarker dynamics validation. (**A**) The standardized residual of biomarkers of 31 incident specimens from 15 WIHS subjects. Each WIHS subject’s first specimen’s days post infection was randomly assigned within the maximum and minimum days post infection (Table 1) and subsequent specimens’ days post infection were based on specimen collection time intervals. This sampling was repeated 10,000 times to obtain each specimen’s biomarker value as a function of days post infection, yielding 10,000 sets of 31 standardized residuals. These standardized residuals were distributed within [−2:2], implying that the WIHS sequence dataset conformed to the *GSI* dynamics inferred from 438 independent specimens presented in Fig. 3A. (**B**) The p-value distribution of 10,000 Anderson-Darling tests. All p values were above 0.28, suggesting that the *GSI* distribution of the WIHS validation dataset is statistically consistent with the model estimate in Fig. 3.

To assess how closely the biomarker dynamics of the WIHS cohort approximate our model estimate, we measured the standardized residual,*e*_*i*_, of each 31 WIHS incident *GSI*, *x*_*i*_.

7 $${e}_{i}={{\rm{\Phi }}}^{-1}({I}_{{x}_{i}}[\alpha (t),\beta (t)]),$$

where Φ is the cumulative distribution function of the standard normal distribution. Here, as defined in Eq. (4), ${I}_{{x}_{i}}[\alpha (t),\beta (t)]$, is the regularized incomplete beta distribution. As WIHS subjects’ last HIV negative and first positive dates are available, the first specimen’s days post infection was assigned randomly within this range (Table 1). Then we assigned the following specimens’ days post infection based on specimen collection intervals. We repeated this random resampling procedure 10,000 times to obtain residuals, *e*_*i*_. All of the standardized residuals were distributed within −2 to 2 (Fig. 7A), suggesting that our validation dataset conforms to the biomarker dynamics estimated by the beta distribution model.

Next we conducted the Anderson-Darling test to evaluate whether the *GSI* distribution of our WIHS test dataset conforms to estimates from our model distribution. Ordering the *GSI* values’ standardized residuals *e*_*i*_ in Eq. (7) from smallest to largest, the test statistics is given by^23, 24^

8 $$Z=A\,(1+\frac{0.75}{{n}_{v}}+\frac{2.25}{{n}_{v}^{2}}),$$

where *n*_*v*_ is the number of test WIHS incident specimens and $A=-{n}_{v}-\frac{1}{{n}_{v}}\sum _{i=1}^{{n}_{v}}(2i-1)[\mathrm{log}({\rm{\Phi }}({e}_{i}))+$ $\,\mathrm{log}(1-{\rm{\Phi }}({e}_{n-i+1}))].$

The Anderson-Darling test p value indicates the likelihood that we observe the measured deviation in Eq. (8), and therefore a higher p value suggests greater conformity of our validation dataset to the model distribution. As described above, we randomly sampled the first specimen’s days from infection and resampled 10,000 times. Figure 7B shows the p-value distribution of 10,000 Anderson-Darling tests; the p-values were above 0.29, we conclude that our validation dataset is statistically consistent with the model distribution.

Serologic assays’ FRRs have been reported to be particularly high in subjects on ART (50–76%) and elite controllers (13–48%)^12^. We evaluated the genomic assay’s FRR for 62 ART-experienced (previously and/or currently on ART) viremic subjects (Tables 1, S1 and S3) and for 16 virally-suppressed ART subjects (Table S4)^25, 26^. The FRR of 62 ART-experienced chronic specimens (Tables 1, S1 and S3) was 3.2% [0–8.1%] while the FRR of 16 virally-suppressed ART specimens was 12.5% [0–31.3%]. As expected, the FRR of the virally suppressed group was considerably higher than that of the ART-experienced chronically viremic subjects. The FRR of 10 elite controller specimens was 40% [10–70%]. Although FRR for the low viral load groups (ART suppressed and elite controllers) was indeed higher than the overall chronic population, where ART did not suppress viral load to undetectable levels the chronic *GSI* signature was maintained and the biomarker score was not significantly affected by ART experiences (Wilcoxon rank sum test, p = 0.67).

## Discussion

The goal of this study was to assess the genomic incidence assay’s FRR and MDRI with a globally representative population to examine the application of this assay in a cross-sectional survey. Compared to the CEPHIA repository, our collection newly covers Asia and Europe but lacks specimens from South America^27^. The sequence similarity biomarker robustly distinguished between incident and chronic infection, providing the best reported FRR (0.33% [0%-0.98%]) and MDRI (420 [361–467] days) and conforming for the first time to the current performance standards^17^.

The MDRI was estimated from the genome similarity index (*GSI*) dynamics of 186 serial and 252 single-time point incident specimens. In the two years following infection, *GSI* decays rapidly in some individuals, and in others is delayed, yielding a bimodal GSI decay spectrum. The flexible Beta distribution was shown to adequately trace not only the average biomarker dynamics but also the breadth of biomarker variability across individuals over time. The *GSI* decay rate was not associated with transmission of multiple founder variants, and was not dependent on subtype, ART experience, risk group, viral load or CD4 + T cell count. Presumably, *GSI* decay speed is influenced by other factors such as immune selection.

The biomarker dynamics were validated using 744 newly sequenced HIV envelope gene segments (HXB2 7134-7499) from 21 longitudinally followed WIHS subjects. Importantly, since both the last HIV negative and first positive test dates were available, we were able to accurately estimate the infection timing interval and thus validate the MDRI estimated from our meta-analyses.

The genomic biomarker was overall not sensitive to viral and host specific factors. Note that serologic assays commonly misclassify chronic subtype D infections as incident at a greater rate (FRR of 9–55%)^12^ than other subtypes, but in this study all three subtype D chronic specimens had *GSI*s below the threshold. Further testing with more subtype D specimens would be required to adequately address the FRR in this group.

The genomic assay’s FRR among virally suppressed subjects were lower than those of serologic assays, however, it was much greater than that of the overall population. On the other hand, where ART did not suppress viral load to undetectable levels, the chronic *GSI* signature was maintained yielding a low FRR of 3.2%. In this assay, ART-experience itself may not cause misclassification of long-standing infection as recent, presumably due to ongoing viral evolution during ART, though the extent of evolution was previously reported to vary across individuals^28, 29^. However, as ART-access increases, accurate incidence determination in populations where low viral load subjects are prevalent still remains a significant challenge.

The FRR of 0.33% allows highly precise incidence determination using the genomic readout of a single blood-draw survey, and the MDRI exceeding 1 year permits incidence to be estimated from a much smaller sample size than existing assays. However, the genomic assay, in a current form, does not meet the optimal performance standards in cost, infrastructure, storage condition, training, regulatory requirement and sample collection method^17^. These factors in the target product profile ought to be evaluated and improved to facilitate application of the genomic assay in cross-sectional surveys.

The genomic assay’s precision recommends it as a reference to evaluate other incidence assays. The genomic incidence assay also has the capacity to detect pre-seroconversion incident infection. Next generation sequencing platforms can be adapted to maximize the genomic assay’s applicability for routine use in cross-sectional settings^19^. Our incidence screen with an envelope gene segment of less than 400 nucleotide bases suggests the feasibility of direct implementation of high-throughput sequencing. Furthermore, the incidence assay’s sequencing approach could be combined with (transmitted) drug resistant surveys to increase the viability of genomic incidence screening.

## Methods

### Meta-analysis cohorts

We first compiled previously published sequences from 274 chronic specimens (Table S1)^19, 30–53^. The chronic specimens were collected at least 2 years after documented HIV infection. A two-year cutoff was implemented based on CEPHIA guidelines^11, 12^. Each specimen’s minimum duration of infection – days from the first HIV positive date, seroconversion, or the first sample collection – is presented in Table SI. Half of the subtyped viruses were subtype B, but subtypes A, C and D were also represented (1%, 3% and 1%, respectively). Eighty percent of samples were from male subjects, 11% from female subjects and sex was not reported for the remaining samples. Subjects’ risk factors were reported to be Men Sex with Men (MSM) (71%), heterosexual (16%), or unknown (13%). The majority of these samples were collected from ART naïve subjects (70%). Eighteen percent of samples were from ART experienced subjects and 12% from subjects whose ART status was not described. Where reported, viral load at sample collection ranged from 437 to 528,140 RNA copies/ml, and the CD4 count ranged from 8 to 1,784 cells/mm^3^.

We next compiled 252 previously published incident specimens’ sequences (Table S2)^36, 39, 54–65^. To best characterize recency, we used Fiebig laboratory staging, not seroconversion date, to estimate each incident specimen’s time since infection along with its 95% confidence interval (17 [13, 28] days for Fiebig stage I, 22 [18, 34] days for II, 25 [22, 37] days for III, 31 [27, 43] days for IV and 101[71, 154] days for V)^66, 67^. Subjects in Fiebig stage VI were not included since the time since infection estimate is open-ended. This group includes subtypes A (2%), B (61%), C (28%), D (2%) and recombinant viruses (6%). Sixty-five percent of samples were collected from male subjects, 24% from female subjects and sex was not reported for the remaining samples. Subjects’ risk factors were described as heterosexual (38%), MSM (27%), Intravenous Drug Use (IDU) (5%) or the remaining unknown. Forty percent of samples were collected from ART naïve subjects and the rest from subjects whose ART status was not described. When reported, viral load ranged from 1,558 to 200,000,000 RNA copies/ml, and the CD4 + T cell count ranged from 6 to 1,040 cells/mm^3^.

Next, we gathered 194 previously published serial specimens from 43 subjects who were followed for up to 3376 days of infection after their first sample was collected within 6 months of transmission (Fiebig stage I-V), as shown in Table S3 ^37, 39, 41, 54, 57, 64, 65, 68^. For example, subject R463F’s first sample was at Fiebig stage IV, and was thus estimated at 31 [27, 43] days post infection. The time since infection of subsequent longitudinal samples was estimated by adding the sampling interval to the first time point estimate, as shown in Table S3. The most abundant subtype in this group was B, but A, C and D were also represented (53%, 13%, 33%, and 1%, respectively). Seventy-four percent of samples were collected from male subjects and the rest from female subjects. These samples were collected from subjects whose risk factor was reported to be MSM (43%), heterosexual (28%), IDU (6%), or the remaining unknown, as presented in Table S3. The majority of these samples were collected from ART naïve subjects (86%) along with 2% from ART experienced subjects and 13% from subjects whose ART status was not reported. When reported, viral load ranged from 49 to 150,000,000 RNA copies/ml, and the CD4 + T cell count ranged from 111 to 1,192 cells/mm^3^.

In addition, we complied published sequences collected from subjects whose viral load was suppressed by ART and from elite controllers, who had been HIV-1 infected for a minimum of 2 years. The ART dataset comprises of 16 specimens from 12 subjects (three female and nine males) in which ART suppressed viral load to an undetectable level for at least 6 months (Table S4)^26, 30, 35^. The CD4 + T cell count in this ART dataset ranged from 223 to 1,174 cell/mm^3^. The group of elite controllers consists of 10 specimens from six chronically infected subjects (two male and four female) who suppressed viremia to less than 50 copies/ml without ART (Table S5 ^30^). The CD4 + T cell count ranged from 383 to 1,125 cells/mm^3^. The sequences of the ART group and elite controllers were isolated not only from plasma but also from resting CD4 + T cells and gut associated lymphoidal tissue (GALT)^26, 30, 35^.

### WIHS cohort

The Women’s Interagency HIV Study (WIHS) was initiated in 1994 and around 2,791 HIV seropositive and 975 seronegative women were enrolled and followed every six months, at six United States sites^69, 70^. Institutional review boards at the WIHS study sites (https://statepi.jhsph.edu/wihs/wordpress/clinical-sites/) approved study procedures and all study participants provided written informed consent. All procedures in this study were performed in accordance with the relevant guidelines and regulations. We newly sequenced a total of 744 HIV-1 envelope gene segments (HXB2 7134-7499) from 21 subjects enrolled in the WIHS. In these subjects, both the last HIV negative and first positive test dates are available, and thus the minimum and maximum days post infection were determined (Table 1). The risk factor of the WIHS subjects was reported to be heterosexual (15%), IDU (46%), or unknown (49%), as presented in Table 1. In 7 subjects ART was initiated during sample collection, and the other 14 subjects remained ART naïve (Fig. 6). While available, viral load ranged from 140 to 1,600,000 RNA copies/ml, and the CD4 + T cell count ranged from 125 to 1,449 cells/mm^3^.

### Sanger sequencing

Sanger sequencing was performed as previously described with several modifications^71, 72^. Viral RNA was extracted from each WIHS plasma specimen (140 μl). Where required, viral RNA was concentrated by pelleting. First, between 0.5 ml and 1 ml of each plasma specimen was centrifuged at 5300 g for 10 minutes at 4 °C. Less than 1 ml of the supernatant liquid was collected and centrifuged at 25,000 g for 2 hours at 4 °C in a 2 ml Sarstedt Microtube. The supernatant was discarded and the RNA was resuspended in 200 μl 1 × phosphate buffered saline (PBS). RNA was extracted using the QIAamp viral RNA Mini Kit (QIAGEN) as per the manufacturer’s instruction with a minor modification. To improve the extraction yield, a two-step elution was performed.

Viral cDNA was synthesized using Superscript III reverse transcriptase (Invitrogen) with the primer envB3out: 5′-TTGCTACTTGTGATTGCTCCATGT-3′, in a reaction volume of 40 µl, comprising 15 µl RNA template, 11 µl first master mix [8.5 µl H_2_O, 2 µl 10 mM dNTP, 0.5 µl primer] and 14 µl of the second master mix [8 µl 5x Buffer, 2 µl 0.1 M DTT, 2 µl RNaseOUT, 2 µl Superscript III reverse transcriptase]. The RNA template with the first master mix was heated at 65 °C for 5 minutes and then placed on ice for 1 minute and spun down. To this mixture, the second master mix was added and then heated at 50 °C for 1 hour. The reaction tube was spun down, then incubated at 55 °C for 1 hour followed by 15 minutes at 70 °C. Subsequently, 2 μl of RNase H was added followed by a final 20 minute incubation at 37 °C. The synthesized cDNA was stored at −20 °C.

The first PCR was conducted using 1 µl of diluted cDNA (from 1:5 to 1:1000) and 19 µl of the master mix [15.5 µl H_2_O, 2 µl 10 × Buffer, 0.4 µl 10 mM dNTP, 0.6 µl 50 mM MgSO_4_, 0.2 µl forward primer (envB5out: 5′-TAGAGCCCTGGAAGCATCCAGGAAG-3′), 0.2 µl reverse primer (envB3out: 5′-TTGCTACTTGTGATTGCTCCATGT-3′), 0.1 µl Platinum High Fidelity Taq Polymerase]. The PCR condition was at 94 °C for 2 minutes, followed by 35 cycles of 94 °C for 15 seconds, 55 °C for 30 seconds, and 68 °C for 5 minutes with a final extension at 68 °C for 10 minutes.

The second PCR was performed using 2 µl of the first PCR product and 18 µl of the master mix [14.5 µl H_2_O, 2 µl 10 × Buffer, 0.4 µl 10 mM dNTP, 0.6 µl 50 mM MgSO_4_, 0.2 µl forward primer (envB5in:5′-TTAGGCATCTCCTATGGCAGGAAGAAG-3′ or For15: 5′-CAGCACAGTACAATGTACACATGGAA-3′), 0.2 µl reverse primer (envB3in:5′-GTCTCGAGATACTGCTCCCACCC-3′ or Rev17: 5′-CCTGGAGCTGTTTAATGCCCCAGAC-3′), 0.1 µl Platinum High Fidelity Taq Polymerase]. The PCR conditions were identical to the first PCR, except that the cycle number was increased to 45. The presence of valid amplicons was verified on 1% Agarose E-gel 96 EtBr (Invitrogen) and GelLogic 212 Pro Imaging System (Carestream). The percentage of wells that contained valid amplicons (% positivity) was recorded for each specimen.

If % positivity exceeds 25%, cDNA was further diluted and the first and second PCRs were repeated to best achieve amplification of only a single cDNA template in each well. As previously described^19^, assuming that the initial cDNA copy number, *Z*, in each well follows a Poisson distribution, U% positivity $(P(Z\ge 1)=U/100)$ yeilds the Poisson Parameter $\lambda =-\mathrm{log}[1-P(Z\ge 1)]=-\mathrm{log}(1-U/100).$ For instance, 25% positivity gives *λ* = 0.29 so the corresponding probability that each well contains more than two copies of cDNA, $P(Z\ge 2)$, is 0.034. Therefore 25% positivity suggests that over 96% of PCRs originated from a single cDNA template.

We can further dilute cDNA using the prior cDNA dilution factor and the corresponding % positivity. As above, the average cDNA copy number per well, the Poisson parameter (*λ*), is a function of % positivity (U), $\lambda =-\mathrm{log}(1-U/100).$ If U% of wells were positive at 1:X dilution, to achieve V% positivity in the next PCR, ideally we need to dilute 1:Y with $Y=X\times \,\mathrm{log}(1-U/100)/\mathrm{log}(1-V/100).$ For example, if 80% of wells were positive at a dilution of 1:10, then the next dilution factor should be 1:56 to best approximate 25% positivity in the next round. We collected a total of 187 amplicons from runs with 25% or less positivity, however, considering specimen availability and associated costs another 557 amplicons were collected from runs with over 25% positivity.

Valid amplicons were then diluted up to 1:10 for Sanger sequencing (BigDye Terminator v3.1, Applied Biosystems). The cycle PCR was performed using 5 µl of diluted (if necessary) cDNA and 5 µl of the master mix [1.5 µl H_2_O, 1.5 µl 5 × Sequencing Buffer, 1 µl of 5 µM primer (NFOR1:5′-TGGCAGTCTAGCAGAAGAAGA-3′), 1 µl BigDye Terminator Ready Reaction Mix]. The cycle PCR condition was at 96 °C for 1 minute, followed by 25 cycles of 96 °C for 10 seconds, 50 °C for 5 seconds, and 60 °C for 4 minutes. The cycle PCR products were purified using the Qiaquick PCR Purification kit (Qiagen) and sequenced with an ABI 3730xl DNA Analyzer (Applied Biosystems). The sequences were marked as non-single genome amplification (SGA) when the chromatogram data showed one or more double peaks in the target region (HXB2 7134-7499). Although around three quarters of the 744 WIHS amplicons were obtained from dilutions yielding greater than 25% positivity, the majority (~93%) were SGA sequences and only 53 sequences (around 7%) were non-SGA sequences. Our experimental protocol was approved by Keck School of Medicine, University of Southern California.

### Sequence cutting and alignment

For our Meta-analysis, we first identified previously published sequences by searching the Los Alamos HIV Sequence database (https://www.hiv.lanl.gov) for sequences with a known timeline, and downloaded the corresponding HIV genomes or segments from Genbank where six or more sequences were reported from a single time point. We aligned both downloaded and newly generated WIHS cohort sequences using ClustalW with manual adjustment. The target envelope segment (HXB2 7134-7499) for GSI analysis, which includes V4 and part of V5, was cut using in-house motif recognition algorithms.

### Subtyping sequences

HIV Blast (https://www.hiv.lanl.gov/content/sequence/BASIC_BLAST/basic_blast.html) and REGA HIV-1 Automated Subtyping tool^73^ (http://dbpartners.stanford.edu:8080/RegaSubtyping/stanford-hiv/typingtool/) were used to identify the subtype of 59 WIHS specimens sequenced in this study and 138 previously published specimens with unknown subtype. The annotated subtype for each specimen is presented in Table 1, Tables S1,S4 and S5. All specimens except those collected from two specimens matched most closely to subtype B sequences.

### Data Availability

The sequences reported in this manuscript are available in GenBank database (accession numbers MF537774-MF538517).

## Electronic supplementary material

Supplementary Information
