# Supplementary material for: The HIV Genomic Incidence Assay Meets False Recency Rate and Mean Duration of Recency Infection Performance Standards
Source: Sci Rep. 2017 Aug 7;7:7480. doi: 10.1038/s41598-017-07490-4 (PMC5547093; doi:10.1038/s41598-017-07490-4)
Supplement: Supplementary file 1 — Supplementary Information [file 41598_2017_7490_MOESM1_ESM.pdf]

SUPPLEMENTARY INFORMATION

The HIV Genomic Incidence Assay Meets False Recency Rate and Mean Duration of Recency Infection Performance Standards

Sung Yong Park, Tanzy M. T. Love, Lucy Reynell, Carl Yu, Tina Manzhu Kang, Kathryn Anastos, Jack DeHovitz, Chenglong Liu, Kord M. Kober, Mardge Cohen, Wendy J. Mack and Ha Youn Lee

Subject (Reference)	Minimum Duration of Infection (days)	Subtype	Risk Behavior	Sex	ART status	Viral Load (RNA copies/ml)	CD4 Count (cells/mm^3)
Bailey [1]							
C61	1,798	B	NS	F	Naïve	19,100	1,261
C62	2,071	B	NS	M	Naïve	33,300	481
C93	1,492	B	NS	M	Naïve	47,270	402
C94	1,886	B	NS	M	Naïve	22,898	351
C96	1,308	B	NS	M	Naïve	12,500	400
C109	882	B	NS	F	Naïve	61,000	222
Yoshida [2]							
7060	1,460	B	MSM	M	Naïve	NS	NS
7015	1,460	B	Heterosexual	NS	Naïve	NS	NS
6826	1,460	B	MSM	M	Naïve	NS	NS
6739	1,460	B	Heterosexual	NS	Naïve	NS	NS
Ren [3]							
CNGX752	1,095	C	NS	NS	Naïve	NS	NS
CNG757	1,095	C	NS	NS	Naïve	NS	NS
CNXJ0031	1,095	R-B/C	NS	NS	Naïve	NS	NS
CNXJ0651	1,095	R-B/C	NS	NS	Naïve	NS	NS
CNXJ1081	1,095	R-B/C	NS	NS	Naïve	NS	NS
CNXJ09277	1,095	C	NS	NS	Naïve	NS	NS
Rong [4]							
ZM185F	787	C	Heterosexual	F	Naïve	NS	NS
	856	C	Heterosexual	F	Naïve	NS	NS
Bunnik [5]							
ACH18969	1,430	B	MSM	M	Naïve	NS	NS
	2,069	B	MSM	M	Experienced	NS	NS
	2,768	B	MSM	M	Experienced	NS	NS
ACH19542	1,308	B	MSM	M	Naïve	NS	NS
	1,916	B	MSM	M	Naïve	NS	NS
	2,616	B	MSM	M	Experienced	NS	NS
	3,559	B	MSM	M	Experienced	NS	NS
ACH19659	913	B	MSM	M	Naïve	NS	NS
	1,886	B	MSM	M	Naïve	NS	NS
	2,920	B	MSM	M	Experienced	NS	NS

B

ACH19768	1,095	B	MSM	M	Experienced	NS	NS
	2,038	B	MSM	M	Experienced	NS	NS
	2,829	B	MSM	M	Experienced	NS	NS
	3,863	B	MSM	M	Experienced	NS	NS
	4,472	B	MSM	M	Experienced	NS	NS
ACH19999	791	B	MSM	M	Naïve	NS	NS
	2,251	B	MSM	M	Naïve	NS	NS
	3,285	B	MSM	M	Experienced	NS	NS
Edo-Matas [6]							
19383	1,886	B	MSM	M	Naïve	NS	NS
	3,255	B	MSM	M	Naïve	NS	NS
	4,045	B	MSM	M	Naïve	NS	NS
19417	2,342	B	MSM	M	Naïve	NS	NS
	3,072	B	MSM	M	Naïve	NS	NS
19559	4,045	B	MSM	M	Naïve	NS	NS
19576	882	B	MSM	M	Naïve	NS	NS
	1,308	B	MSM	M	Naïve	NS	NS
19828	1,430	B	MSM	M	Naïve	NS	NS
19858	1,278	B	MSM	M	Naïve	NS	NS
	3,437	B	MSM	M	Naïve	NS	NS
19922	2,494	B	MSM	M	Naïve	NS	NS
	4,106	B	MSM	M	Naïve	NS	NS
Van-Gils [7]							
19298	1,034	B	MSM	M	Naïve	NS	NS
	1,460	B	MSM	M	Naïve	NS	NS
	2,190	B	MSM	M	Experienced	NS	NS
	2,647	B	MSM	M	Experienced	NS	NS
19554	1,430	B	MSM	M	Naïve	NS	NS
	2,069	B	MSM	M	Naïve	NS	NS
	2,525	B	MSM	M	Experienced	NS	NS
	3,255	B	MSM	M	Experienced	NS	NS
19663	1,430	B	MSM	M	Naïve	NS	NS
	2,768	B	MSM	M	Naïve	NS	NS
	3,377	B	MSM	M	Naïve	NS	NS
	4,259	B	MSM	M	Naïve	NS	NS
19642	882	B	MSM	M	Naïve	NS	NS

B

	1,491	B	MSM	M	Naïve	NS	NS
	2,251	B	MSM	M	Naïve	NS	NS
	3,285	B	MSM	M	Naïve	NS	NS
	3,985	B	MSM	M	Naïve	NS	NS
	4,320	B	MSM	M	Naïve	NS	NS
Gnanakaran [8]							
MCST4474	3,468	B	MSM	M	Naïve	29,500	634
RHGA1581	3,468	B	MSM	M	Naïve	25,901	571
STCO5453	730	B	MSM	M	Naïve	67,964	796
WARO5662	913	B	Heterosexual	F	Naïve	16,758	598
Keele [9]							
CRPE4571	730	B	Heterosexual	F	Naïve	21,917	67
FOJO4081	5,718	B	MSM	M	Naïve	73,677	408
LAHA4867	730	B	MSM	M	Naïve	53,500	610
MCRO3633	2,738	B	Heterosexual	F	Naïve	13,175	305
OLLA4645	760	B	Heterosexual	F	Naïve	382,000	150
SAMI4303	1,430	B	MSM	M	Naïve	116,000	1,012
TALA4022	2,525	B	MSM	M	Naïve	228,200	8
WICU4248	2,160	B	MSM	M	Naïve	8,424	564
YOMI4024	2,220	B	MSM	M	Naïve	14,178	486
UNC3405	2,890	B	MSM	M	Naïve	29,100	495
UNC5057	1,764	B	Heterosexual	F	Naïve	141,000	277
UNC5417	1,156	B	Heterosexual	M	Naïve	48,000	340
UNC5479	1,673	B	Heterosexual	F	Naïve	15,400	382
UNC5539	1,855	B	MSM	M	Naïve	46,600	355
UNC5769	973	B	MSM	M	Naïve	18,100	466
UNC5791	1,582	B	Heterosexual	F	Naïve	65,566	262
UNC7092	5,475	B	Heterosexual	F	Naïve	64,700	333
UNC2009	1,430	B	MSM	M	Naïve	56,000	343
UNC4295	2,707	B	MSM	M	Naïve	74,800	495
UNC4484	2,251	B	Heterosexual	F	Naïve	7,580	244
UNC4911	1,886	B	Heterosexual	F	Naïve	13,500	438
UNC5283	1,521	B	Heterosexual	F	Naïve	43,100	455
UNC5548	1,308	B	Heterosexual	F	Naïve	1,560	337

B

UNC5734	1,369	B	Heterosexual, IDU	F	Naïve	12,800	317
UNC5799	4,015	B	MSM	M	Naïve	81,400	211
UNC6064	1,491	B	MSM	M	Naïve	10,400	468
SC02	1,095	B	Heterosexual	M	Naïve	76,615	NS
SC03	2,525	B	Heterosexual	M	Naïve	528,140	121
SC05	1,977	B	Heterosexual	M	Naïve	19,514	NS
SC13	973	B	Heterosexual	M	Naïve	12,054	NS
SC24	1,399	B	Heterosexual	M	Naïve	178,753	NS
SC25	1,004	B	Heterosexual	F	Naïve	218,551	NS
TT31P	730	B	Heterosexual	F	Naïve	19,909	525
Herbeck [10]							
PIC51550	3,285	B	MSM	M	Naïve	28,610	NS
Herbeck [10] Liu [11]							
PIC1365	3,653	B	MSM	M	Naïve	NS	NS
Daniels [12]							
Pat-1	3,285	B	NS	M	Naïve	NS	220
Pat-2	5,476	B	NS	M	Naïve	NS	170
Doria-Rose [13]							
CAP256	1,233	C	Heterosexual	F	Naïve	NS	NS
Wu [14]							
1	3,650	B	NS	NS	Naïve	14,650	1,160
	5,840	B	NS	NS	Naïve	10,526	568
	7,665	B	NS	NS	Experienced	19,260	545
18	2,920	B	NS	NS	Naïve	24,648	462
45	4,015	B	NS	NS	Naïve	11,416	727
	5,840	B	NS	NS	Naïve	9,129	638
	6,935	B	NS	NS	Naïve	5,153	686
N26	7,665	B	NS	NS	Naïve	35,912	387
N90	8395	B	NS	NS	Naïve	8,216	912
Blish [15], Piantadosi [16]							
QA413	1,007	A	Heterosexual	F	Naïve	NS	296
	1,146	A	Heterosexual	F	Naïve	NS	NS
	1,346	A	Heterosexual	F	Naïve	NS	NS
Geels [17]							

B

H671	1,430	B	MSM	M	Naïve	NS	NS
Sturdevant [18]							
9018	873	B	NS	NS	Experienced	89,125	453
	980	B	NS	NS	Experienced	95,499	505
9040	753	B	NS	NS	Naïve	275,422	279
SSemwanga [19]							
PP1_F1	883	D	Heterosexual	F	NS	NS	NS
PP1_F3	2,312	D	Heterosexual	F	NS	NS	NS
PP1_F4	2,570	A/D	Heterosexual	F	NS	NS	NS
PP1_M	2,824	D	Heterosexual	M	NS	NS	NS
Skar [20]							
45755	2,555	B	NS	M	Naïve	832	NS
	2,556	B	NS	M	Naïve	794	NS
	2,557	B	NS	M	Naïve	1220	NS
	2,565	B	NS	M	Naïve	563	NS
	2,572	B	NS	M	Naïve	518	NS
	2,579	B	NS	M	Naïve	450	NS
	2,586	B	NS	M	Naïve	600	NS
	3,076	B	NS	M	Naïve	NS	NS
Ping [21]							
CAP61	835	C	Heterosexual	F	NS	NS	NS
DU123	1,702	C	Heterosexual	F	NS	NS	NS
Mukhopadhyay [22]							
991566	2,920	B/C	NS	NS	Naïve	NS	720
Malherbe [23]							
VC10014	1,037	B	Heterosexual		Naïve	NS	NS
	1,310	B	Heterosexual		Naïve	NS	NS
VC20013	774	B	NS		Naïve	NS	NS
Evering [24]							
NCN1	1,813	B	MSM	M	Experienced	65,600	591
NCN2	5,265	B	MSM	M	Naïve	749	802
NCN3	1,488	B	MSM	M	Experienced	8,620	687
NCN4	6,521	B	MSM	M	Naïve	13,900	790
NCN5	2,291	B	MSM	M	Naïve	19,700	420
NCN6	4,166	B	MSM	M	Experienced	18,900	418
ANI1	4,688	B	MSM	M	Naïve	5,320	743
ANI2	6,440	B	MSM	M	Experienced	29,800	220

B

ANI3	1,405	B	MSM	M	Naïve	1,890	819
ANI4	1,709	B	MSM	M	Naïve	38,600	237
ANI5	806	B	MSM	M	Naïve	9,210	244
ANI6	1,817	B	MSM	M	Experienced	13,000	329
ANI7	6,284	B	MSM	M	Experienced	50,100	466
MND1	2,282	B	MSM	M	Naïve	6,250	398
MND2	1,780	B	Heterosexual	F	Experienced	44,900	320
Shankarappa [25], Jensen [26]							
p1	1,044	B	MSM	M	Naïve	13,243	845
	1,369	B	MSM	M	Naïve	53,211	755
	1,544	B	MSM	M	Naïve	13,614	463
	1,851	B	MSM	M	Naïve	11,455	484
	1,993	B	MSM	M	Naïve	17,258	534
	2,081	B	MSM	M	Naïve	NS	524
	2,354	B	MSM	M	Naïve	103,039	505
	2,435	B	MSM	M	Naïve	206,063	147
	2,657	B	MSM	M	Naïve	53,333	44
	2,851	B	MSM	M	Naïve	107,152	59
	2,982	B	MSM	M	Naïve	20,230	27
	3,194	B	MSM	M	Naïve	41,020	42
p2	927	B	MSM	M	Naïve	6,067	1,579
	1,205	B	MSM	M	Naïve	3,532	1,556
	1,566	B	MSM	M	Naïve	220,293	1,784
	1,843	B	MSM	M	Naïve	46,774	1,667
	2,070	B	MSM	M	Naïve	34,995	1,710
	2,230	B	MSM	M	Naïve	48,753	1,654
	2,420	B	MSM	M	Naïve	202,768	1,112
	2,595	B	MSM	M	Naïve	345,939	693
	2,763	B	MSM	M	Naïve	269,153	470
	3,139	B	MSM	M	Naïve	83,176	178
	3,829	B	MSM	M	Experienced	163,682	100
p3	785	B	MSM	M	Naïve	6,237	686
	920	B	MSM	M	Naïve	7,709	576
	1,292	B	MSM	M	Naïve	1,282	672
	1,668	B	MSM	M	Naïve	17,783	734
	2,033	B	MSM	M	Naïve	2,466	624

B

	2,219	B	MSM	M	Naïve	19,953	367
	2,424	B	MSM	M	Experienced	117,490	254
	2,935	B	MSM	M	Experienced	59,429	NS
p4	821	B	MSM	M	NS	16,218	513
	989	B	MSM	M	NS	3,715	529
	1,164	B	MSM	M	NS	72,277	411
	1,278	B	MSM	M	NS	NS	387
	1,453	B	MSM	M	NS	91201	278
	1,599	B	MSM	M	NS	62087	370
	1,796	B	MSM	M	NS	4667	280
	1,971	B	MSM	M	NS	209411	278
	2,154	B	MSM	M	NS	254097	189
	2,321	B	MSM	M	NS	130017	47
	2,712	B	MSM	M	NS	232809	8
p5	843	B	MSM	M	Naïve	66,834	742
	1,029	B	MSM	M	Naïve	16,904	692
	1,223	B	MSM	M	Naïve	101,625	500
	1,318	B	MSM	M	Naïve	32,137	869
	1,497	B	MSM	M	Naïve	21,330	615
	1,701	B	MSM	M	Naïve	26,485	498
	1,894	B	MSM	M	Naïve	77,268	334
	2,070	B	MSM	M	Naïve	182,390	241
	2,456	B	MSM	M	Experienced	41,591	18
p6	730	B	MSM	M	Naïve	57,943	526
	902	B	MSM	M	Naïve	53,211	404
	1,080	B	MSM	M	Naïve	133,968	371
	1,263	B	MSM	M	Naïve	228,034	291
	1,445	B	MSM	M	Experienced	240,991	311
	1,628	B	MSM	M	Experienced	240,991	276
	2,208	B	MSM	M	Experienced	263,027	24
p7	763	B	MSM	M	Naïve	6,823	739
	931	B	MSM	M	Naïve	21,577	685
	1,343	B	MSM	M	Naïve	21,979	460
	1,518	B	MSM	M	Experienced	9,162	543
	1,690	B	MSM	M	Experienced	124,738	494
	1,872	B	MSM	M	Experienced	50,234	537
	2,252	B	MSM	M	Experienced	42,855	373

B

	2,438	B	MSM	M	Experienced	76,384	237
p8	876	B	MSM	M	Naïve	9,441	545
	1,066	B	MSM	M	Naïve	7,194	448
	1,234	B	MSM	M	Naïve	35,075	591
	1,409	B	MSM	M	Naïve	21,577	463
	1,617	B	MSM	M	Naïve	22,594	359
	1,799	B	MSM	M	Naïve	35,400	410
	1,989	B	MSM	M	Naïve	113,763	278
	2,143	B	MSM	M	Naïve	28,445	259
	2,456	B	MSM	M	Experienced	17,783	55
	2,887	B	MSM	M	Experienced	23,335	87
p9	1,172	B	MSM	M	Naïve	8,204	625
	1,905	B	MSM	M	Experienced	528	1,115
	2,621	B	MSM	M	Experienced	4,477	1,295
	3,172	B	MSM	M	Experienced	12,735	947
	3,362	B	MSM	M	Experienced	29,717	NS
	3,544	B	MSM	M	Experienced	73,451	549
	3,712	B	MSM	M	Experienced	73,451	258
	3,909	B	MSM	M	Experienced	201,837	612
	4,084	B	MSM	M	Experienced	333,426	202
p10	785	B	MSM	M	NS	802	1,588
	975	B	MSM	M	NS	575	1,615
	1,336	B	MSM	M	NS	3192	1,681
	1,511	B	MSM	M	NS	NS	1,740
	1,891	B	MSM	M	NS	NS	1,062
	2,051	B	MSM	M	NS	NS	1,260
	2,241	B	MSM	M	NS	NS	1,140
	2,424	B	MSM	M	NS	NS	1,019
	2,602	B	MSM	M	NS	11,041	1,095
	2,792	B	MSM	M	NS	NS	953
	3,362	B	MSM	M	NS	NS	1,030
	3,548	B	MSM	M	NS	NS	774
	4,081	B	MSM	M	NS	NS	885
	4,263	B	MSM	M	NS	NS	805
	4,464	B	MSM	M	NS	759	808
	4,668	B	MSM	M	NS	437	789
	4,811	B	MSM	M	NS	564	779

B

p11	883	B	MSM	M	Naïve	741	831
	1,270	B	MSM	M	Naïve	490	840
	1,763	B	MSM	M	Naïve	468	1,230
	2,128	B	MSM	M	Naïve	1,778	1,118
	3,030	B	MSM	M	Naïve	NS	823
Park [27]		B					
PL7408	749	B	MSM	M	Naive	507	840
IJ7234	2,984	B	MSM	M	Naive	5,599	364
QZ8149	3,216	B	Heterosexual	F	Naive	15,000	542
LE2707	2,407	B	Heterosexual	F	Naive	1,394	607
CX7332	5,464	B	Heterosexual	M	Experienced	389,081	63
KI6633	2,428	B	Heterosexual	M	Experienced	460,004	115
CF6610	3,710	B	MSM	M	Experienced	17,001	634
Liao [28]							
CH505	924	C	Heterosexual	M	Naive	51,554	294
	1,092	C	Heterosexual	M	Naive	110,230	339

Table S1. Chronic specimens. NS, not stated in original publication; Naïve, subject has not received ART before this time point; Experienced, patient is currently or has previously received ART; MSM, men who have sex with men; IDU, intravenous drug user.

B

Subject (Reference)	Estimated Days Post Infection	Fiebig Stage	Subtype	Risk Behavior	Sex	ART status	Viral Load (RNA copies/ml)	CD4 Count (cells/mm^3)
Baalwa [29], Yue [30]								
190049	19.5 [13, 34]	I/II	D	Heterosexual	F	Naïve	38,266,666	NS
191084	31 [27, 43]	IV	A	Heterosexual	F	Naïve	34,600	NS
191727	31 [27, 43]	IV	D	Heterosexual	F	Naïve	15,200	NS
191845	31 [27, 43]	IV	A	Heterosexual	F	Naïve	31,200	NS
191859	17 [13, 28]	I	D	Heterosexual	F	Naïve	53,334	NS
191882	17 [13, 28]	I	D	Heterosexual	M	Naïve	15,378	NS
191947	17 [13, 28]	I	R-A/D	Heterosexual	M	Naïve	5,466	NS
191982	17 [13, 28]	I	A/D	Heterosexual	F	Naïve	7,156	NS
Chen [31]								
BJOX23	31 [27, 43]	IV	R-A/E	MSM	M	Naïve	4,380,000	NS
BJOX35	19.5 [13, 34]	I/II	B	MSM	M	Naïve	11,600	NS
BJOX19	19.5 [13, 34]	I/II	R-B/C	MSM	M	Naïve	330,000	NS
BJOX20	19.5 [13, 34]	I/II	B	MSM	M	Naïve	64,700	NS
BJOX15	19.5 [13, 34]	I/II	R-A/E	MSM	M	Naïve	1,180,000	NS
BJOX03	19.5 [13, 34]	I/II	B	MSM	M	Naïve	2,150,000	NS
BJOX22	31 [27, 43]	IV	B	MSM	M	Naïve	322,000	NS
BJOX05	19.5 [13, 34]	I/II	R-A/E	MSM	M	Naïve	30,211	NS
BJOX06	31 [27, 43]	IV	B	MSM	M	Naïve	498,947	NS
BJOX25	19.5 [13, 34]	I/II	R-A/E	MSM	M	Naïve	1,740,000	NS
BJOX09	31 [27, 43]	IV	R-A/E	MSM	M	Naïve	77,600	NS
BJOX18	31 [27, 43]	IV	R-A/E	MSM	M	Naïve	16,200	NS
BJOX28	19.5 [13, 34]	I/II	R-A/E	MSM	M	Naïve	70,200	NS
BJOX10	19.5 [13, 34]	I/II	R-A/E	MSM	M	Naïve	19,300	NS
BJOX12	101 [71, 154]	V	R-A/E	MSM	M	Naïve	3,330	NS
BJOX29	101 [71, 154]	V	R-B/C	MSM	M	Naïve	7,440	NS
BJOX07	101 [71, 154]	V	B	MSM	M	Naïve	1,558	NS
BJOX11	101 [71, 154]	V	R-B/C	MSM	M	Naïve	11,900	NS
BJOX31	101 [71, 154]	V	R-A/E	MSM	M	Naïve	14,700	NS
BJOX47	101 [71, 154]	V	B	MSM	M	Naïve	4,480	NS
BJOX14	101 [71, 154]	V	B	MSM	M	Naïve	2,480,000	NS
BJOX41	101 [71, 154]	V	B	MSM	M	Naïve	6,080	NS
BJOX46	101 [71, 154]	V	B	MSM	M	Naïve	18,700	NS
Masharsky [32]								

B

R163	19.5 [13, 34]	I/II	A	IDU	F	Naïve	316,228	NS
R392	31 [27, 43]	IV	A	IDU	M	Naïve	501,187	NS
R497	31 [27, 43]	IV	A	IDU	M	Naïve	398,107	NS
R575	31 [27, 43]	IV	A	IDU	M	Naïve	630,957	NS
Manak [33]								
BP00002	22 [18, 34]	II	C	NS	M	Naïve	553,000	NS
BP00055	22 [18, 34]	II	B	NS	M	Naïve	13,200,000	NS
BP00008	101 [71, 154]	V	C	NS	M	Naïve	471,000	NS
BP00010	101 [71, 154]	V	C	NS	M	Naïve	112,000	NS
BP00067	101 [71, 154]	V	B	NS	M	Naïve	199,000	NS
Keele [9]								
1006-11	25 [22, 37]	III	B	NS	M	NS	1,600,000	NS
1054-07	22 [18, 34]	II	B	NS	M	NS	320,000	NS
6240-08	22 [18, 34]	II	B	NS	M	NS	120,000	NS
6244-13	22 [18, 34]	II	B	NS	M	NS	274,000	NS
9010-09	22 [18, 34]	II	B	NS	F	NS	146,954	NS
9014-01	22 [18, 34]	II	B	NS	M	NS	25,680	NS
9015-07	22 [18, 34]	II	B	NS	M	NS	500,000	NS
9017-01	22 [18, 34]	II	B	NS	M	NS	32,390	NS
9020-20	22 [18, 34]	II	B	NS	F	NS	69,238	NS
9021-14	22 [18, 34]	II	B	NS	M	NS	143,379	NS
9023-21	22 [18, 34]	II	B	NS	M	NS	>500,000	NS
9024-12	22 [18, 34]	II	B	NS	NS	NS	>500,000	NS
9025-11	22 [18, 34]	II	B	NS	M	NS	>500,000	NS
9028-07	22 [18, 34]	II	B	NS	F	NS	>500,000	NS
9032-08	25 [22, 37]	III	B	NS	M	NS	40,815	NS
9033-16	25 [22, 37]	III	B	NS	M	NS	454,553	NS
9075-03	22 [18, 34]	II	B	NS	NS	NS	360,000	NS
9077-12	22 [18, 34]	II	B	NS	NS	NS	158,300	NS
9079-09	22 [18, 34]	II	B	NS	NS	NS	148,320	NS
61792-03	22 [18, 34]	II	B	NS	NS	NS	3,700,000	NS
62130-04	22 [18, 34]	II	B	NS	NS	NS	600,000	NS
62357-14	22 [18, 34]	II	B	NS	M	NS	302,000	NS
62995-05	17 [13, 28]	I	B	NS	NS	NS	75,000	NS
63054-04	22 [18, 34]	II	B	NS	NS	NS	1,153,260	NS
63396-07	22 [18, 34]	II	B	NS	NS	NS	420,000	NS
PRB926-04	22 [18, 34]	II	B	NS	NS	NS	756,060	NS

B

PRB931-06	25 [22, 37]	III	B	NS	NS	NS	2,000,000	NS
PRB956-04	22 [18, 34]	II	B	NS	NS	NS	600,000	NS
PRB958-06	25 [22, 37]	III	B	NS	NS	NS	678,900	NS
PRB959-02	22 [18, 34]	II	B	NS	NS	NS	>2,000,000	NS
SC05	22 [18, 34]	II	B	Heterosexual	M	NS	9,980,952	NS
SC11	22 [18, 34]	II	B	Heterosexual	F	NS	1,917,073	NS
SC20	31 [27, 43]	IV	B	Heterosexual	M	NS	2,789,313	NS
SC45	22 [18, 34]	II	B	Heterosexual	M	NS	6,318,529	NS
TRJO4551	22 [18, 34]	II	B	MSM	M	NS	8,121,951	NS
TT29P	22 [18, 34]	II	B	Heterosexual	M	NS	14,756,436	NS
WITO4160	22 [18, 34]	II	B	Heterosexual	M	NS	325,064	NS
Z05	22 [18, 34]	II	B	Heterosexual	M	NS	3,950,942	NS
Z20	25 [22, 37]	III	B	MSM	M	NS	13,466,851	NS
Z32	31 [27, 43]	IV	B	MSM	M	NS	5,788,591	NS
Z34	25 [22, 37]	III	B	Bisexual	M	NS	2,501,242	NS
1018-10	25 [22, 37]	III	B	NS	M	NS	270,000	NS
1053-07	25 [22, 37]	III	B	NS	M	NS	1,400,000	NS
6248-07	25 [22, 37]	III	B	NS	M	NS	87,700,000	NS
1001-07	25 [22, 37]	III	B	NS	F	NS	210,000	NS
9022-09	25 [22, 37]	III	B	NS	M	NS	>500,000	NS
TT35P	22 [18, 34]	II	B	Heterosexual	M	NS	1,849,301	NS
63358-04	22 [18, 34]	II	B	NS	NS	NS	260,000	NS
9031-19	31 [27, 43]	IV	B	NS	M	NS	237,938	NS
9029-12	22 [18, 34]	II	B	NS	M	NS	>500,000	NS
12007-04	22 [18, 34]	II	B	NS	NS	NS	75,815	NS
1012-11	25 [22, 37]	III	B	NS	M	NS	410,000	NS
SC22	22 [18, 34]	II	B	Heterosexual	F	NS	14,304,820	NS
9030-15	22 [18, 34]	II	B	NS	M	NS	>500,000	NS
SC31	31 [27, 43]	IV	B	Heterosexual	F	NS	4,449,612	NS
700010058	25 [22, 37]	III	B	NS	M	NS	394,649	NS
Z33	22 [18, 34]	II	B	Bisexual	M	NS	158,603	NS
Z31	22 [18, 34]	II	B	MSM	M	NS	6,507,075	NS
63068-05	22 [18, 34]	II	B	NS	NS	NS	100,000	NS
TT27P	31 [27, 43]	IV	B	Heterosexual	M	NS	1,489,202	NS
62615-03	22 [18, 34]	II	B	NS	NS	NS	2,200,000	NS
Z35	31 [27, 43]	IV	B	MSM	M	NS	2,281,415	NS
9026-07	25 [22, 37]	III	B	NS	NS	NS	211,703	NS

B

9076-08	25 [22, 37]	III	B	NS	NS	NS	410,000	NS
SC33	22 [18, 34]	II	B	Heterosexual	M	NS	3,277,559	NS
BORI0637	22 [18, 34]	II	B	MSM	M	NS	2,400,000	NS
PRB957-06	22 [18, 34]	II	B	NS	NS	NS	>2,000,000	NS
12008-09	22 [18, 34]	II	B	NS	NS	NS	>2,000,000	NS
SC42	31 [27, 43]	IV	B	Heterosexual	M	NS	1,380,000	NS
9019-03	101 [71, 154]	V	B	NS	F	NS	571,000	NS
REJO4541	101 [71, 154]	V	B	Heterosexual	M	NS	722,349	NS
THRO4156	101 [71, 154]	V	B	MSM	M	NS	5,413,140	NS
TT28P	101 [71, 154]	V	B	Heterosexual	M	NS	7,468,346	NS
TT34P	101 [71, 154]	V	B	Heterosexual	M	NS	3,050,125	NS
Z02	101 [71, 154]	V	B	Heterosexual	M	NS	296,111	NS
Z13	101 [71, 154]	V	B	Heterosexual	M	NS	53,941	NS
Z23	101 [71, 154]	V	B	Bisexual	M	NS	210,521	NS
Z27	101 [71, 154]	V	B	Heterosexual	M	NS	178,125	NS
RHPA4259	101 [71, 154]	V	B	Heterosexual	F	NS	1,458,354	NS
63215-03	101 [71, 154]	V	B	NS	NS	NS	550,000	NS
SC51	101 [71, 154]	V	B	Heterosexual	M	Naïve	4,229,703	NS
MEMI4948	101 [71, 154]	V	B	MSM	M	NS	22,686	NS
700010019	101 [71, 154]	V	B	IDU	F	NS	741,499	NS
Z18	101 [71, 154]	V	B	MSM	M	NS	2,845,823	NS
CAAN5342	101 [71, 154]	V	B	MSM	M	NS	608,560	NS
Z30	101 [71, 154]	V	B	Heterosexual	F	NS	1,408,114	NS
Z16	101 [71, 154]	V	B	MSM	M	NS	2,375,617	NS
Z03	101 [71, 154]	V	B	MSM	M	NS	726,859	NS
701010016	101 [71, 154]	V	B	NS	M	NS	1,802	NS
Z29	101 [71, 154]	V	B	Bisexual	M	NS	507,401	NS
Abrahams [34]								
0114	31 [27, 43]	IV	C	Heterosexual	M	NS	99,000,000	NS
0334	19.5 [13, 34]	I/II	C	Heterosexual	M	NS	22,000,000	NS
0393	31 [27, 43]	IV	C	Heterosexual	F	NS	12,000,000	NS
0478	19.5 [13, 34]	I/II	C	Heterosexual	M	NS	2,166,667	NS
0595	31 [27, 43]	IV	C	Heterosexual	F	NS	1,006,864	NS
0626	31 [27, 43]	IV	C	Heterosexual	M	NS	711,061	NS
0665	31 [27, 43]	IV	C	Heterosexual	M	NS	67,728	NS
0682	19.5 [13, 34]	I/II	C	Heterosexual	M	NS	17,960	NS
0985	19.5 [13, 34]	I/II	C	Heterosexual	M	NS	2,894,737	NS

B

1086	19.5 [13, 34]	I/II	C	Heterosexual	M	NS	1,409,208	NS
1172	19.5 [13, 34]	I/II	C	Heterosexual	M	NS	4,805,921	NS
1176	19.5 [13, 34]	I/II	C	Heterosexual	M	NS	200,000,000	NS
1196	19.5 [13, 34]	I/II	C	Heterosexual	M	NS	30,500,000	NS
1335	31 [27, 43]	IV	C	Heterosexual	M	NS	576,282	NS
1373	19.5 [13, 34]	I/II	C	Heterosexual	F	NS	3,221,979	NS
1394	19.5 [13, 34]	I/II	C	Heterosexual	M	NS	7,341,772	NS
2010	31 [27, 43]	IV	C	Heterosexual	M	NS	1,203,333	NS
2052	19.5 [13, 34]	I/II	C	Heterosexual	F	NS	339,475	NS
2060	19.5 [13, 34]	I/II	C	Heterosexual	M	NS	1,530,364	NS
2103	19.5 [13, 34]	I/II	C	Heterosexual	M	NS	152,364	NS
703010010	25 [22, 37]	III	C	Heterosexual	M	NS	408,727	NS
703010131	25 [22, 37]	III	C	Heterosexual	M	NS	437,369	NS
703010159	22 [18, 34]	II	C	Heterosexual	F	NS	73,453	NS
703010228	31 [27, 43]	IV	C	Heterosexual	M	NS	335,000	NS
704010083	25 [22, 37]	III	C	Heterosexual	F	NS	>750,000	NS
704809221	19.5 [13, 34]	I/II	C	Heterosexual	M	NS	>750,000	NS
CAP37	31 [27, 43]	IV	C	Heterosexual	F	NS	248,000	NS
CAP63	25 [22, 37]	III	C	Heterosexual	F	NS	3,210,000	NS
CAP69	19.5 [13, 34]	I/II	C	Heterosexual	F	NS	15,300	NS
CAP129	31 [27, 43]	IV	C	Heterosexual	F	NS	1,800,000	NS
CAP177	19.5 [13, 34]	I/II	C	Heterosexual	F	NS	359,000	NS
CAP188	19.5 [13, 34]	I/II	C	Heterosexual	F	NS	13,800,000	NS
CAP200	31 [27, 43]	IV	C	Heterosexual	F	NS	398,000	NS
CAP217	31 [27, 43]	IV	C	Heterosexual	F	NS	3,260,000	NS
CAP221	19.5 [13, 34]	I/II	C	Heterosexual	F	NS	24,300	NS
CAP222	19.5 [13, 34]	I/II	C	Heterosexual	F	NS	69,700	NS
CAP225	25 [22, 37]	III	C	Heterosexual	F	NS	63,600,000	NS
CAP237	25 [22, 37]	III	C	Heterosexual	F	NS	1,020,000	NS
089	101 [71, 154]	V	C	Heterosexual	M	NS	83,844	NS
703010054	101 [71, 154]	V	C	Heterosexual	M	NS	13,936	NS
703010193	101 [71, 154]	V	C	Heterosexual	M	NS	46,724	NS
704010042	101 [71, 154]	V	C	Heterosexual	M	NS	133,000	NS
704810053	101 [71, 154]	V	C	Heterosexual	F	NS	25,400	NS
705010015	101 [71, 154]	V	C	Heterosexual	F	NS	47,900	NS
705010026	101 [71, 154]	V	C	Heterosexual	F	NS	8,971	NS
705010078	101 [71, 154]	V	C	Heterosexual	M	NS	255,907	NS

B

CAP8	101 [71, 154]	V	C	Heterosexual	F	NS	207,000	NS
CAP84	101 [71, 154]	V	C	Heterosexual	F	NS	559,000	NS
CAP85	101 [71, 154]	V	C	Heterosexual	F	NS	621,000	NS
CAP136	101 [71, 154]	V	C	Heterosexual	F	NS	85,300	NS
CAP174	101 [71, 154]	V	C	Heterosexual	F	NS	40,000	NS
CAP206	101 [71, 154]	V	C	Heterosexual	F	NS	196,000	NS
CAP220	101 [71, 154]	V	C	Heterosexual	F	NS	2,070	NS
CAP224	101 [71, 154]	V	C	Heterosexual	F	NS	1,348,000	NS
CAP260	101 [71, 154]	V	C	Heterosexual	F	NS	17,600	NS
Bar [35]								
HDNDRPI029	31 [27, 43]	IV	B	IDU	F	NS	21,878	440
HDNDRPI034	25 [22, 37]	III	B	IDU	M	NS	75,857,758	240
CQLDR03	101 [71, 154]	V	B	IDU	M	NS	102,329	NS
HDNDRPI001	101 [71, 154]	V	B	IDU	M	NS	870,964	690
HDNDRPI032	101 [71, 154]	V	B	IDU	M	NS	3,388	1,040
HTM319	101 [71, 154]	V	B	IDU	M	NS	26,915	520
HTM385	101 [71, 154]	V	B	IDU	M	NS	234,423	406
Heipertz [36]								
306159	19.5 [13, 34]	I/II	B	NS	NS	Naïve	NS	NS
306344	19.5 [13, 34]	I/II	B	NS	NS	Naïve	NS	NS
306376	19.5 [13, 34]	I/II	B	NS	NS	Naïve	NS	NS
306512	19.5 [13, 34]	I/II	B	NS	NS	Naïve	NS	NS
306517	19.5 [13, 34]	I/II	B	NS	NS	Naïve	NS	NS
Li [37]								
INME0632	22 [18, 34]	II	B	MSM	M	Naïve	2,217,670	739
HOBR0961	22 [18, 34]	II	B	MSM	M	Naïve	599,238	794
4013171	31 [27, 43]	IV	B	MSM	M	Naïve	3,700,000	213
4013211	25 [22, 37]	III	B	MSM	M	Naïve	19,900,000	846
4013226	22 [18, 34]	II	B	MSM	M	Naïve	26,700,000	175
4013240	22 [18, 34]	II	B	MSM	M	Naïve	2,240,000	297
4013242	31 [27, 43]	IV	B	MSM	M	Naïve	5,790,000	251
4013291	101 [71, 154]	V	B	MSM	M	Naïve	1,490,000	179
4013296	22 [18, 34]	II	B	MSM	M	Naïve	8,050,000	395
4013321	22 [18, 34]	II	B	MSM	M	Naïve	6,250,000	407
4013327	31 [27, 43]	IV	B	MSM	M	Naïve	8,720,000	248
4013383	22 [18, 34]	II	B	MSM	M	Naïve	584,000	531
4013396	31 [27, 43]	IV	B	MSM	M	Naïve	1,600,000	581

B

4013419	22 [18, 34]	II	B	MSM	M	Naïve	21,200,000	226
4013440	22 [18, 34]	II	B	MSM	M	Naïve	>100,000	205
4013446	25 [22, 37]	III	B	MSM	M	Naïve	>100,000	438
4013448	22 [18, 34]	II	B	MSM	M	Naïve	28,600,000	536
701010055	22 [18, 34]	II	B	MSM	M	Naïve	31,513,812	432
701010068	31 [27, 43]	IV	B	MSM	M	Naïve	3,714,386	109
AD17	22 [18, 34]	II	B	MSM	M	Naïve	47,600,000	NS
AD75	22 [18, 34]	II	B	MSM	M	Naïve	21,400,000	NS
AD83	101 [71, 154]	V	B	MSM	M	Naïve	448,000	NS
700010106	22 [18, 34]	II	B	MSM	M	Naïve	84,545,454	277
701010027	101 [71, 154]	V	B	MSM	M	Naïve	194,744	542
700010246	31 [27, 43]	IV	B	MSM	M	Naïve	4,395,721	1,012
AD77	101 [71, 154]	V	B	MSM	M	Naïve	130,000	NS
701010108	101 [71, 154]	V	B	MSM	M	Naïve	14,711	592
700010238	101 [71, 154]	V	B	MSM	M	Naïve	596,908	587
Parrish[38, 39]								
705010067	19.5 [13, 34]	I/II	C	Heterosexual	F	Naïve	639,000	NS
20258279	31 [27, 43]	IV	C	NS	F	Naïve	281,838	NS
1245045	19.5 [13, 34]	I/II	C	NS	M	Naïve	234,068	NS
2833264	19.5 [13, 34]	I/II	C	NS	M	Naïve	234,423	NS
2935054	19.5 [13, 34]	I/II	C	NS	M	Naïve	>10,000,000	NS
19157834	19.5 [13, 34]	I/II	C	NS	M	Naïve	275,423	NS
20927783	19.5 [13, 34]	I/II	C	NS	F	Naïve	1,886	NS
21197826	19.5 [13, 34]	I/II	C	NS	F	Naïve	343,923	NS
21283649	19.5 [13, 34]	I/II	C	NS	M	Naïve	3,180	NS
Gnanakaran [8]								
701010043	22 [18, 34]	II	B	NS	F	Naïve	5,532	NS
Z64	31 [27, 43]	IV	B	Heterosexual	M	Naïve	11,503,872	6
SC24	23.5 [18, 37]	II/III	B	Heterosexual	M	Naïve	523,183	NS
SC50	23.5 [18, 37]	II/III	B	Heterosexual	M	Naïve	259,747	NS
FASH1057	22 [18, 34]	II	B	Heterosexual	F	Naïve	21,783,636	NS
700010224	101 [71, 154]	V	B	NS	M	Naïve	300,000	NS
701010092	101 [71, 154]	V	B	NS	M	Naïve	207,143	NS
Z75	101 [71, 154]	V	B	MSM	M	Naïve	170,826	241
Z78	101 [71, 154]	V	B	MSM	M	Naïve	750,001	NS
Z91	101 [71, 154]	V	B	MSM	M	Naïve	37,706	541

B

Z92	101 [71, 154]	V	B	MSM	M	Naïve	7,633,876	NS
Z95	101 [71, 154]	V	B	Heterosexual	M	Naïve	59,603	808
Nofemela [40]								
98	101 [71, 154]	V	C	Heterosexual	F	Naïve	5,330	NS
304	101 [71, 154]	V	C	Heterosexual	F	Naïve	>750,001	NS
556	101 [71, 154]	V	C	Heterosexual	F	Naïve	43,300	NS
49	25 [22, 37]	III	R-A/C/D	Heterosexual	F	Naïve	>750,001	NS
532	19.5 [13, 34]	I/II	R-A/D	Heterosexual	F	Naïve	>750,001	NS
390	101 [71, 154]	V	C	Heterosexual	F	Naïve	>750,001	NS

Table S2. Incident specimens with Fiebig stage I, II, III, IV, and V.

B

Subject	Estimated Days Post Infection	Days from First Sample	Fiebig Stage	Subtype	Risk Behaviour	Sex	ART status	Viral Load (RNA copies/ml)	CD4 T cell Count (cells/mm^3)
Baalwa [29], Yue [30]									
191647	22 [18, 34]	0	II	D	Heterosexual	M	Naïve	242,222	NS
	149 [145, 161]	127	-	D	Heterosexual	M	Naïve	4,470	518
R463F	31 [27, 43]	0	IV	A	Heterosexual	F	Naïve	150,000,000	NS
	38 [34, 50]	7	-	A	Heterosexual	F	Naïve	3,980,000	324
	79 [75, 91]	48	-	A	Heterosexual	F	Naïve	332,000	427
	198 [194, 210]	167	-	A	Heterosexual	F	Naïve	398,000	368
	267 [263, 279]	236	-	A	Heterosexual	F	Naïve	246,000	344
	352 [348, 364]	321	-	A	Heterosexual	F	Naïve	167,054	375
R880F	25 [22, 37]	0	III	A	Heterosexual	F	Naïve	430,843	NS
	88 [85, 100]	63	-	A	Heterosexual	F	Naïve	2,410	483
	172 [169, 184]	147	-	A	Heterosexual	F	Naïve	49	742
	256 [253, 268]	231	-	A	Heterosexual	F	Naïve	49	NS
	338 [335, 350]	313	-	A	Heterosexual	F	Naïve	62	704
9004SS	31 [27, 43]	0	IV	A	Heterosexual	M	Naïve	29,921	NS
	60 [56, 72]	29	-	A	Heterosexual	M	Naïve	12,323	1,192
Herbeck [10], Liu [11]									
PIC11286	101 [71, 154]	0	V	B	MSM	M	Naïve	2,804,000	NS
	106 [76,159]	5	-	B	MSM	M	Naïve	54,160	NS
	114 [84,167]	13	-	B	MSM	M	Naïve	29,180	NS
PIC38051	17 [13,28]	0	I	B	MSM	M	Naïve	620,820	NS
	21 [17,32]	4	-	B	MSM	M	Naïve	2,498,840	NS
PIC38417	101 [71,154]	0	V	B	MSM	M	Naïve	472,810	NS
	109 [79,162]	8	-	B	MSM	M	Naïve	169,580	NS
	116 [86,169]	15	-	B	MSM	M	Naïve	117,310	NS
	129 [99,182]	28	-	B	MSM	M	Naïve	71,230	NS
	159 [129,212]	58	-	B	MSM	M	Naïve	30,650	NS
	192 [162,245]	91	-	B	MSM	M	Naïve	32,430	NS
	220 [190, 273]	119	-	B	MSM	M	Naïve	24,740	NS
	276 [246,329]	175	-	B	MSM	M	Naïve	19,460	NS
	323 [293,376]	222	-	B	MSM	M	Naïve	14,610	NS
PIC55751	101 [71,154]	0	V	B	MSM	M	Naïve	128,814	NS

B

	115 [85, 168]	14	-	B	MSM	M	Naïve	8,469	NS
	3,426 [3396, 3479]	3325	-	B	MSM	M	Naïve	40,600	NS
	3,477 [3447, 3530]	3376	-	B	MSM	M	Naïve	22,190	NS
PIC71101	17 [13, 28]	0	I	B	MSM	M	Naïve	377,060	NS
	23 [19, 34]	6	-	B	MSM	M	Naïve	1,912,200	NS
	31 [27, 42]	14	-	B	MSM	M	Naïve	312,200	NS
	54 [50, 65]	37	-	B	MSM	M	Naïve	32,630	NS
	78 [74, 89]	61	-	B	MSM	M	Naïve	232,050	NS
	106 [102, 117]	89	-	B	MSM	M	Naïve	109,950	NS
	137 [133, 148]	120	-	B	MSM	M	Naïve	45,590	NS
	191 [187, 202]	174	-	B	MSM	M	Naïve	105,870	NS
PIC83747	17 [13, 28]	0	I	B	MSM	M	Naïve	923	NS
	23 [19, 34]	6	-	B	MSM	M	Naïve	1,049,870	NS
	31 [27, 42]	14	-	B	MSM	M	Naïve	26,785,000	NS
	53 [49, 64]	36	-	B	MSM	M	Naïve	665,680	NS
	59 [55, 70]	42	-	B	MSM	M	Naïve	668,660	NS
	67 [63, 79]	50	-	B	MSM	M	Naïve	124,000	NS
	80 [76, 91]	63	-	B	MSM	M	Naïve	254,300	NS
	109 [105, 120]	92	-	B	MSM	M	Naïve	151,000	NS
	162 [159, 173]	145	-	B	MSM	M	Naïve	242,940	NS
	221 [217, 232]	204	-	B	MSM	M	Naïve	60,950	NS
	284 [280, 295]	267	-	B	MSM	M	Naïve	51,120	NS
	361 [357, 372]	344	-	B	MSM	M	Naïve	49,600	NS
PIC90770	17 [13, 28]	0	I	B	MSM	M	Naïve	469,830	NS
	25 [21, 36]	8	-	B	MSM	M	Naïve	1600000	NS
	29 [25, 40]	12	-	B	MSM	M	Naïve	42,810	NS
	85 [81, 96]	68	-	B	MSM	M	Naïve	12,200	NS
	113 [109, 124]	96	-	B	MSM	M	Naïve	14,170	NS
	142 [138, 153]	125	-	B	MSM	M	Naïve	18,210	NS
	210 [206, 221]	193	-	B	MSM	M	Naïve	70,780	NS
PIC1362	17 [13, 28]	0	I	B	MSM	M	Naïve	8,240,000	876
	31 [27, 42]	14	-	B	MSM	M	Naïve	NS	NS
	59 [55, 70]	42	-	B	MSM	M	Naïve	NS	NS
	122 [118, 133]	105	-	B	MSM	M	Naïve	NS	NS
	164 [160, 175]	147	-	B	MSM	M	Naïve	NS	NS
	199 [195, 210]	182	-	B	MSM	M	Naïve	NS	NS
	353 [349, 364]	336	-	B	MSM	M	Naïve	NS	NS

B

	590 [586, 601]	573	-	B	MSM	M	Naïve	NS	NS
	778 [774, 789]	761	-	B	MSM	M	Naïve	NS	NS
	835 [831, 846]	818	-	B	MSM	M	Naïve	NS	NS
	1,044 [1040, 1055]	1,027	-	B	MSM	M	Naïve	NS	NS
	1,256 [1252, 1267]	1,239	-	B	MSM	M	Naïve	NS	NS
	1,500 [1496, 1511]	1,483	-	B	MSM	M	Naïve	NS	NS
Parrish [39]									
703010200	19.5 [13, 34]	0	I/II	C	Heterosexual	M	NS	165,501	NS
	26.5 [20, 41]	7	IV	C	Heterosexual	M	NS	128,677	111
	417.5 [411, 432]	398	-	C	Heterosexual	M	NS	27,882	NS
	704.5 [698, 719]	685	-	C	Heterosexual	M	NS	18,300	NS
Liu [11], Keele [9], Salazar-Gonzalez [41], Abrahams [34]									
CAP045	19.5 [13, 34]	0	I/II	C	Heterosexual	F	Naive	12,500	NS
	40.5 [34, 55]	21	-	C	Heterosexual	F	Naive	236,000	974
	369.5 [363, 384]	350	-	C	Heterosexual	F	Naive	556	1,030
CAP210	19.5 [13, 34]	0	I/II	C	Heterosexual	F	Naive	468,000	332
	89.5 [83,104]	70	-	C	Heterosexual	F	Naive	50,100	543
	159.5 [153,174]	140	-	C	Heterosexual	F	Naive	NS	NS
	187.5 [181, 202]	168	-	C	Heterosexual	F	Naive	20,400	475
CH040	22 [18, 34]	0	II	B	MSM	M	Naive	2,197,248	NS
	67 [63, 79]	45	-	B	MSM	M	Naive	298,026	NS
	133 [129, 145]	111	-	B	MSM	M	Naive	NS	NS
	203 [199, 215]	181	-	B	MSM	M	Naive	NS	NS
	434 [430, 446]	412	-	B	MSM	M	Naive	NS	NS
CH042	31 [27, 43]	0	IV	C	NS	M	Naive	181,000	NS
	52 [48, 64]	21	-	C	NS	M	Naive	201,000	318
	91 [87, 103]	60	-	C	NS	M	Naive	128,000	350
	203 [199, 215]	172	-	C	NS	M	Naive	102,000	357
	455 [451, 467]	424	-	C	NS	M	Naive	86,400	295
	707 [703, 719]	676	-	C	NS	M	Naive	267,000	349
CH058	22 [18, 34]	0	II	B	MSM	M	Naive	92,581	NS
	58 [54, 70]	36	-	B	MSM	M	Naive	394,649	NS
	98 [94, 110]	76	-	B	MSM	M	Naive	NS	NS

B

CH077	19.5 [13, 34]	0	I/II	B	MSM	M	Naive	NS	NS
	33.5 [27, 48]	14	-	B	MSM	M	Naive	NS	NS
	51.5 [45, 66]	32	-	B	MSM	M	Naive	NS	NS
	178.5 [172, 193]	159	-	B	MSM	M	Naive	NS	NS
CH131	19.5 [13, 34]	0	I/II	C	NS	M	Naive	411,873	NS
	40.5 [34, 55]	21	-	C	NS	M	Naive	7,764	NS
	47.5 [41, 62]	28	-	C	NS	M	Naive	10,655	NS
	53.5 [47, 68]	34	-	C	NS	M	Naive	53,169	NS
	82.5 [76, 97]	63	-	C	NS	M	Naive	45,697	NS
	110.5 [104, 125]	91	-	C	NS	M	Naive	15,424	NS
	194.5 [188, 209]	175	-	C	NS	M	Naive	50,709	310
	292.5 [286, 307]	273	-	C	NS	M	Naive	27,446	300
	352.5 [346, 367]	333	-	C	NS	M	Naive	44,488	298
	689.5 [683, 704]	670	-	C	NS	M	Naive	17,807	214
CH159	19.5 [13, 34]	0	I/II	C	Heterosexual	F	Naive	17,805	NS
	27.5 [21, 42]	8		C	Heterosexual	F	Naive	587,457	NS
	31.5 [25, 46]	12		C	Heterosexual	F	Naive	570,786	NS
	41.5 [35, 56]	22	-	C	Heterosexual	F	Naive	7,101	NS
	48.5 [42, 63]	29	-	C	Heterosexual	F	Naive	13,566	NS
	75.5 [69, 90]	56	-	C	Heterosexual	F	Naive	9,397	NS
	104.5 [98, 119]	85	-	C	Heterosexual	F	Naive	71,474	463
	321.5 [315, 336]	302	-	C	Heterosexual	F	Naive	3,722	448
	667.5 [661, 682]	648	-	C	Heterosexual	F	Naive	<400	432
	1151.5 [1144.5, 1165]	1132	-	C	Heterosexual	F	Naive	10,161	347
CH162	25 [22, 37]	0	III	C	NS	M	Naive	>10,000,000	NS
	46 [43, 58]	21	-	C	NS	M	Naive	18,260	NS
	102 [99, 114]	77	-	C	NS	M	Naive	3,625	NS
	204 [201, 216]	179	-	C	NS	M	Naive	102,793	533
	463 [460, 475]	438	-	C	NS	M	Naive	295,950	204
CH164	25 [22, 37]	0	III	C	NS	M	Naive	23,600	700
	39 [36, 51]	14	-	C	NS	M	Naive	NS	NS
	81 [78, 93]	56	-	C	NS	M	Naive	7,720	NS
	194 [191, 206]	169	-	C	NS	M	Naive	12,700	449
	445 [442, 456]	420	-	C	NS	M	Naive	750,000	NS
CH185	19.5 [13, 34]	0	I/II	C	Heterosexual	F	Naive	20,449	NS
	44.5 [38, 59]	25	-	C	Heterosexual	F	Naive	85,443	NS

B

	86.5 [80, 101]	67	-	C	Heterosexual	F	Naive	79,706	NS
	199.5 [193, 214]	180	-	C	Heterosexual	F	Naive	46,772	NS
	435.5 [429, 450]	416	-	C	Heterosexual	F	Naive	234,010	311
CH198	19.5 [13, 34]	0	I/II	C	Heterosexual	M	Naive	14,950,000	NS
	30.5 [24, 45]	11	-	C	Heterosexual	M	Naive	301,401	599
	79.5 [73, 94]	60	-	C	Heterosexual	M	Naive	2,433	NS
	466.5 [460, 481]	447	-	C	Heterosexual	M	Naive	3,476	NS
CH256	19.5 [13, 34]	0	I/II	C	Heterosexual	F	Naive	62,060	NS
	47.5 [41, 62]	28	-	C	Heterosexual	F	Naive	46,342	NS
	82.5 [76, 97]	63	-	C	Heterosexual	F	Naive	35,328	NS
	191.5 [185, 206]	172	-	C	Heterosexual	F	Naive	13,484	NS
	445.5 [439, 460]	426	-	C	Heterosexual	F	Naive	6,183	601
	703.5 [697, 718]	684	-	C	Heterosexual	F	Naive	28,066	531
CH470	31 [27, 43]	0	IV	B	NS	M	Naive	84,193	NS
	44 [40, 56]	13	-	B	NS	M	Naive	264,882	324
	72 [68, 84]	41	-	B	NS	M	Naive	27,991	522
	100 [96, 112]	69	-	B	NS	M	Naive	27,869	NS
	205 [201, 217]	174	-	B	NS	M	Naive	28,456	554
CAP239	101 [71, 154]	0	V	C	Heterosexual	F	Naive	95,800	845
	220 [190, 273]	119	-	C	Heterosexual	F	Naive	22,400	984
CH607	31 [27, 43]	0	IV	B	MSM	M	Experienced	8,009	NS
	45 [41, 57]	14		B	MSM	M	Experienced	NS	NS
	52 [48, 64]	21		B	MSM	M	Experienced	24,545	NS
Masharsky [32]									
K84	31 [27, 43]	0	IV	A	IDU	M	NS	251,189	NS
	55 [51, 55]	24	V	A	IDU	M	NS	NS	NS
R053	31 [27, 43]	0	IV	A	IDU	F	NS	398,107	NS
	39 [35, 51]	8	V	A	IDU	F	NS	NS	NS
R526	19.5 [13, 34]	0	I/II	A	IDU	M	NS	501,187	NS
	84.5 [78, 99]	65	-	A	IDU	M	NS	NS	NS
H386	31 [27, 43]	0	IV	A	IDU	F	NS	630,957	NS
	40 [36, 52]	9	-	A	IDU	F	NS	NS	NS
H408	25 [22, 37]	0	III	A	IDU	M	NS	NS	NS
	38 [35, 50]	13	IV	A	IDU	M	NS	251,189	NS

B

H410	25 [22, 37]	0	III	A	IDU	M	NS	630,957	NS
	38 [35, 50]	13	IV	A	IDU	M	NS	NS	NS
Keele [9]									
SUMA0874	22 [18, 34]	0	II	B	MSM	M	Naive	NS	NS
	23 [19, 35]	1	II	B	MSM	M	Naive	939,260	NS
	31 [27, 43]	9	III	B	MSM	M	Naive	701,640	NS
	38 [34, 50]	16	IV	B	MSM	M	Naive	106,543	NS
	52 [48, 64]	30	V	B	MSM	M	Naive	14,780	NS
WEAU0575	22 [18, 34]	0	II	B	MSM	M	Naive	216,415	NS
	30 [26, 42]	8	IV	B	MSM	M	Naive	355,360	NS
	37 [33, 49]	15	IV	B	MSM	M	Naive	100,877	NS
	51 [47, 63]	29	V	B	MSM	M	Naive	34,737	NS
	219 [215, 231]	197	VI	B	MSM	M	Naive	90,109	NS
1059	22 [18, 34]	0	II	B	NS	M	Naive	82,204	NS
	26 [22, 38]	4	II	B	NS	M	Naive	> 100,000	NS
	29 [25, 41]	7	III	B	NS	M	Naive	2,800,000	NS
1058	17 [13, 28]	0	I	B	NS	M	Naïve	2,737	NS
	20 [16, 31]	3	II	B	NS	M	Naïve	26,162	NS
	27 [23, 38]	10	IV	B	NS	M	Naive	550,000	NS
6247	17 [13, 28]	0	I	B	NS	F	NS	23,858	NS
	19 [15, 30]	2	II	B	NS	F	NS	124,000	NS
TT31P	22 [18, 34]	0	II	B	Heterosexual	F	NS	21,826,347	NS
	33 [29, 45]	11	IV	B	Heterosexual	F	NS	59,189	NS
1051	17 [13, 28]	0	I	B	NS	F	NS	13,000	NS
	27 [23, 38]	10	II	B	NS	F	NS	280,000	NS
1056	17 [13, 28]	0	I	B	NS	M	NS	7,000	NS
	20 [16, 31]	3	II	B	NS	M	NS	65,000	NS
	24 [20, 35]	7	II	B	NS	M	NS	140,000	NS

Table S3. Longitudinal specimens from 43 subjects

B

Subject	Minimum Duration of Infection (days)	Subtype	Risk Behavior	Sex	Viral Load (RNA copies/ml)	CD4 Count (cells/mm^3)	Source
Bailey [1]							
H22	5,936	B	NS	F	<50	1,174	Plasma
	6,119	B	NS	F	<50	NS	Resting CD4+ T cells
H23	1,218	B	NS	M	<50	571	Plasma
	1,401	B	NS	M	<50	NS	Resting CD4+ T cells
H28	4,869	B	NS	M	<50	534	Plasma
H135	4,901	B	NS	F	<50	384	Plasma
	4,718	B	NS	F	<50	NS	Resting CD4+ T cells
H154	6,576	B	NS	M	<50	223	Plasma
	6,576	B	NS	M	<50	223	Resting CD4+ T cells
H9	7,153	B	NS	M	<50	NS	Resting CD4+ T cells
H25	1,432	B	NS	M	<50	NS	Resting CD4+ T cells
H26	2,072	B	NS	M	<50	NS	Resting CD4+ T cells
H148	5,997	B	NS	F	<50	NS	Resting CD4+ T cells
Bruner [42]							
2286	4,471	B	NS	M	< 50	NS	Resting CD4+ T cells
CP03	5,900	B	NS	M	< 50	NS	Resting CD4+ T cells
Evering [24]							
IIA	761	B	MSM	M	< 50	903	GALT

Table S4. Virally suppressed ART subjects

B

Subject (Reference)	Minimum Duration of Infection (days)	Subtype	Risk Behavior	Sex	ART status	Viral Load (RNA copies/ml)	CD4 Count (cells/mm^3)	Source
Bailey [1]								
ES2	6,331	B	NS	F	Naïve	<50	383	Plasma
ES4	2,770	B	NS	F	Naïve	<50	837	Plasma
	2,770	B	NS	F	Naïve	<50	837	Resting CD4+ T cells
ES5	4,962	B	NS	F	Naïve	<50	704	Resting CD4+ T cells
ES6	4,200	B	NS	F	Naïve	<50	733	Resting CD4+ T cells
ES7	3,654	B	NS	M	Naïve	<50	1,125	Plasma
	3,654	B	NS	M	Naïve	<50	1,125	Resting CD4+ T cells
ES9	1,552	B	NS	M	Naïve	<50	800	Plasma
	1,675	B	NS	M	Naïve	<50	NS	Plasma
	1,859	B	NS	M	Naïve	<50	NS	Resting CD4+ T cells

Table S5. Elite Controllers

B

REFERENCES

1 Bailey, J. R., Williams, T. M., Siliciano, R. F. & Blankson, J. N. Maintenance of viral suppression in HIV-1-infected HLA-B*57+ elite suppressors despite CTL escape mutations. *J Exp Med* **203**, 1357-1369 (2006).

2 Yoshida, I. *et al.* Change of positive selection pressure on HIV-1 envelope gene inferred by early and recent samples. *PLoS One* **6**, e18630 (2011).

3 Ren, C. *et al.* Cross-neutralizing antibody profile of Chinese HIV-1-infected individuals and the viral envelope features from elite neutralizers. *J Acquir Immune Defic Syndr* **67**, 472-480 (2014).

4 Rong, R. *et al.* Escape from autologous neutralizing antibodies in acute/early subtype C HIV-1 infection requires multiple pathways. *PLoS Pathog* **5**, e1000594 (2009).

5 Bunnik, E. M., Pisas, L., van Nuenen, A. C. & Schuitemaker, H. Autologous neutralizing humoral immunity and evolution of the viral envelope in the course of subtype B human immunodeficiency virus type 1 infection. *J Virol* **82**, 7932-7941 (2008).

6 Edo-Matas, D. *et al.* Impact of CCR5delta32 host genetic background and disease progression on HIV-1 intrahost evolutionary processes: efficient hypothesis testing through hierarchical phylogenetic models. *Mol Biol Evol* **28**, 1605-1616 (2011).

7 van Gils, M. J. *et al.* Rapid escape from preserved cross-reactive neutralizing humoral immunity without loss of viral fitness in HIV-1-infected progressors and long-term nonprogressors. *J Virol* **84**, 3576-3585 (2010).

8 Gnanakaran, S. *et al.* Recurrent signature patterns in HIV-1 B clade envelope glycoproteins associated with either early or chronic infections. *PLoS Pathog* **7**, e1002209 (2011).

9 Keele, B. F. *et al.* Identification and characterization of transmitted and early founder virus envelopes in primary HIV-1 infection. *Proc Natl Acad Sci U S A* **105**, 7552-7557 (2008).

10 Herbeck, J. T. *et al.* Demographic processes affect HIV-1 evolution in primary infection before the onset of selective processes. *J Virol* **85**, 7523-7534 (2011).

11 Liu, Y. *et al.* Selection on the human immunodeficiency virus type 1 proteome following primary infection. *J Virol* **80**, 9519-9529 (2006).

12 Daniels, R. S., Wilson, P., Patel, D., Longhurst, H. & Patterson, S. Analysis of full-length HIV type 1 env genes indicates differences between the virus infecting T cells and dendritic cells in peripheral blood of infected patients. *AIDS Res Hum Retroviruses* **20**, 409-413 (2004).

13 Doria-Rose, N. A. *et al.* Developmental pathway for potent V1V2-directed HIV-neutralizing antibodies. *Nature* **509**, 55-62 (2014).

14 Wu, X. *et al.* Selection pressure on HIV-1 envelope by broadly neutralizing antibodies to the conserved CD4-binding site. *J Virol* **86**, 5844-5856 (2012).

15 Blish, C. A. *et al.* Human immunodeficiency virus type 1 superinfection occurs despite relatively robust neutralizing antibody responses. *J Virol* **82**, 12094-12103 (2008).

16 Piantadosi, A., Chohan, B., Chohan, V., McClelland, R. S. & Overbaugh, J. Chronic HIV-1 infection frequently fails to protect against superinfection. *PLoS Pathog* **3**, e177 (2007).
17 Geels, M. J. *et al.* Identification of sequential viral escape mutants associated with altered T-cell responses in a human immunodeficiency virus type 1-infected individual. *J Virol* **77**, 12430-12440 (2003).
18 Sturdevant, C. B. *et al.* Compartmentalized replication of R5 T cell-tropic HIV-1 in the central nervous system early in the course of infection. *PLoS Pathog* **11**, e1004720 (2015).
19 Ssemwanga, D. *et al.* Multiple HIV-1 infections with evidence of recombination in heterosexual partnerships in a low risk Rural Clinical Cohort in Uganda. *Virology* **411**, 113-131 (2011).
20 Skar, H. *et al.* Daily sampling of an HIV-1 patient with slowly progressing disease displays persistence of multiple env subpopulations consistent with neutrality. *PLoS One* **6**, e21747 (2011).
21 Ping, L. H. *et al.* Comparison of viral Env proteins from acute and chronic infections with subtype C human immunodeficiency virus type 1 identifies differences in glycosylation and CCR5 utilization and suggests a new strategy for immunogen design. *J Virol* **87**, 7218-7233 (2013).
22 Mukhopadhyay, S., Ringe, R., Patil, A., Paranjape, R. & Bhattacharya, J. Characterization of circulating HIV type 1 env genes in plasma of two antiretroviral-naive slow progressing patients with broad neutralizing antibody response with evidence of recombination. *AIDS Res Hum Retroviruses* **28**, 739-745 (2012).
23 Malherbe, D. C. *et al.* Envelope variants circulating as initial neutralization breadth developed in two HIV-infected subjects stimulate multiclade neutralizing antibodies in rabbits. *J Virol* **88**, 12949-12967 (2014).
24 Evering, T. H. *et al.* Single genome analysis reveals genetic characteristics of Neuroadaptation across HIV-1 envelope. *Retrovirology* **11**, 65 (2014).
25 Shankarappa, R. *et al.* Consistent viral evolutionary changes associated with the progression of human immunodeficiency virus type 1 infection. *J Virol* **73**, 10489-10502 (1999).
26 Jensen, M. A. *et al.* Improved coreceptor usage prediction and genotypic monitoring of R5-to-X4 transition by motif analysis of human immunodeficiency virus type 1 env V3 loop sequences. *J Virol* **77**, 13376-13388 (2003).
27 Park, S. Y. *et al.* Developing high-throughput HIV incidence assay with pyrosequencing platform. *J Virol* **88**, 2977-2990 (2014).
28 Liao, H. X. *et al.* Co-evolution of a broadly neutralizing HIV-1 antibody and founder virus. *Nature* **496**, 469-476 (2013).
29 Baalwa, J. *et al.* Molecular identification, cloning and characterization of transmitted/founder HIV-1 subtype A, D and A/D infectious molecular clones. *Virology* **436**, 33-48 (2013).
30 Yue, L. *et al.* Transmitted virus fitness and host T cell responses collectively define divergent infection outcomes in two HIV-1 recipients. *PLoS Pathog* **11**, e1004565 (2015).

31 Chen, Y. *et al.* Comprehensive Characterization of the Transmitted/Founder env Genes From a Single MSM Cohort in China. *J Acquir Immune Defic Syndr* **69**, 403-412 (2015).
32 Masharsky, A. E. *et al.* A substantial transmission bottleneck among newly and recently HIV-1-infected injection drug users in St Petersburg, Russia. *J Infect Dis* **201**, 1697-1702 (2010).
33 Manak, M. *et al.* Pilot studies for development of an HIV subtype panel for surveillance of global diversity. *AIDS Res Hum Retroviruses* **28**, 594-606 (2012).
34 Abrahams, M. R. *et al.* Quantitating the multiplicity of infection with human immunodeficiency virus type 1 subtype C reveals a non-poisson distribution of transmitted variants. *J Virol* **83**, 3556-3567 (2009).
35 Bar, K. J. *et al.* Wide variation in the multiplicity of HIV-1 infection among injection drug users. *J Virol* **84**, 6241-6247 (2010).
36 Heipertz, R. A., Jr. *et al.* Molecular epidemiology of early and acute HIV type 1 infections in the United States Navy and Marine Corps, 2005-2010. *AIDS Res Hum Retroviruses* **29**, 1310-1320 (2013).
37 Li, H. *et al.* High Multiplicity Infection by HIV-1 in Men Who Have Sex with Men. *PLoS Pathog* **6**, e1000890 (2010).
38 Parrish, N. F. *et al.* Transmitted/founder and chronic subtype C HIV-1 use CD4 and CCR5 receptors with equal efficiency and are not inhibited by blocking the integrin alpha4beta7. *PLoS Pathog* **8**, e1002686 (2012).
39 Parrish, N. F. *et al.* Phenotypic properties of transmitted founder HIV-1. *Proc Natl Acad Sci U S A* **110**, 6626-6633 (2013).
40 Nofemela, A. *et al.* Defining the human immunodeficiency virus type 1 transmission genetic bottleneck in a region with multiple circulating subtypes and recombinant forms. *Virology* **415**, 107-113 (2011).
41 Salazar-Gonzalez, J. F. *et al.* Genetic identity, biological phenotype, and evolutionary pathways of transmitted/founder viruses in acute and early HIV-1 infection. *J Exp Med* **206**, 1273-1289 (2009).
42 Bruner, K. M. *et al.* Defective proviruses rapidly accumulate during acute HIV-1 infection. *Nat Med* **22**, 1043-1049 (2016).

B
